# From macro to micro: a combined bioluminescence‐fluorescence approach to monitor bacterial localization

**DOI:** 10.1111/1462-2920.15296

**Published:** 2021-01-22

**Authors:** Riccardo Soldan, Nattapong Sanguankiattichai, Marcel Bach‐Pages, Indra Bervoets, Wei E. Huang, Gail M. Preston

**Affiliations:** ^1^ Department of Plant Sciences University of Oxford Oxford UK; ^2^ Department of Bioengineering Sciences Vrije Universiteit Brussel Brussels Belgium; ^3^ Department of Engineering University of Oxford Oxford UK

## Abstract

Bacterial bioluminescence is widely used to study the spatiotemporal dynamics of bacterial populations and gene expression *in vivo* at a population level but cannot easily be used to study bacterial activity at the level of individual cells. In this study, we describe the development of a new library of mini‐Tn7‐*lux* and *lux*::*eyfp* reporter constructs that provide a wide range of *lux* expression levels, and which combine the advantages of both bacterial bioluminescence and fluorescent proteins to bridge the gap between macro‐ and micro‐scale imaging techniques. We demonstrate that a dual bioluminescence‐fluorescence approach using the *lux* operon and eYFP can be used to monitor bacterial movement in plants both macro‐ and microscopically and demonstrate that *Pseudomonas syringae* pv *phaseolicola* can colonize the leaf vascular system and systemically infect leaves of common bean (*Phaseolus vulgaris*). We also show that bacterial bioluminescence can be used to study the impact of plant immune responses on bacterial multiplication, viability and spread within plant tissues. The constructs and approach described in this study can be used to study the spatiotemporal dynamics of bacterial colonization and to link population dynamics and cellular interactions in a wide range of biological contexts.

## Introduction

In recent years, advances in DNA sequencing technology and molecular biology techniques have provided important new insights into the composition and functions of host‐associated bacteria for both plants (Bulgarelli *et al*., 2012; Rybakova *et al*., 2017) and animals (Turnbaugh *et al*., 2008; Le Chatelier *et al*., 2013). However, although these methods are of pivotal importance in furthering our understanding of the biological processes governing microbial communities and their functions, they often provide limited insight into the spatio‐temporal aspects of host–microbe and microbe–microbe interactions. Such methods typically aggregate information across spatially distributed populations, with information being collected for a limited number of samples and timepoints (Prosser, 2010; Fierer and Ladau, 2012). However, the output of an interaction between a bacterial pathogen and its host (animal or plant) greatly depends on the type of tissue that is colonized, the local microenvironment (spatial relationship) and the movement of the pathogen over time within the host (spatiotemporal relationship) (Lamichhane *et al*., 2015). The homogenisation of a limited number of biological samples to isolate macromolecules or to enumerate microbial cells can obscure variation in microbial physiology at the macro‐ and micro‐scale. For this reason, visualizing and studying bacteria in their environment continue to be important (Propheter and Hooper, 2015) and considerable effort has been invested into developing tools to allow visualization of bacteria in the host environment (Seleem *et al*., 2008; Monteiro *et al*., 2012).

For example, the spatial relationships of microbe–microbe interactions can be studied using fluorescent *in situ* hybridization (FISH) coupled with confocal laser scanning microscopy (CLSM) (Earle *et al*., 2015; Soldan *et al*., 2019). The main advantage of FISH is the observation of spatial relationships between multiple members of a microbial community without necessarily knowing *a priori* the composition of the community itself. However, FISH requires fixed samples, thus preventing the study of real‐time interactions. For this reason, bacteria tagged with fluorescent proteins (e.g. YFP, GFP) have been extensively used to visualize the spatial and temporal relationships of host–microbe interactions for both detrimental (Godfrey *et al*., 2010; Cerutti *et al*., 2017; Donati *et al*., 2018; Planas‐Marquès *et al*., 2020) and beneficial (Compant *et al*., 2005; Fan *et al*., 2011; Mitter *et al*., 2017) relationships.

However, while fluorescent proteins allow researchers to investigate bacterial colonization at micro‐scale resolution, they are not suitable for macro‐scale resolution studies, such as analyses of systemic infection. This is partially due to the relatively weak fluorescence of labelled bacteria and the autofluorescence of the host (Wang *et al*., 2007). This can result in a labour‐intensive process of collecting a series of host samples that can be visualized with fluorescence microscopy in an attempt to localize labelled bacteria without knowing *a priori* their location. Light‐sheet microscopy, such as the ultramicroscope from La Vision Bio Tec, allows imaging of tissue samples up to centimetres in size (Reynaud *et al*., 2015), but even with this technology, imaging a whole plant organ, such as a leaf, would not be feasible for many plant species. Moreover, this specialized equipment is not commonly accessible to many laboratories. Wang *et al*. (2007) proposed that a brighter green‐fluorescent protein variant, called GFPuv, encoded in a broad‐host‐range high‐copy number plasmid could allow monitoring of bacterial disease at whole‐plant level using UV light. However, with a few notable exceptions (Rodríguez‐Moreno *et al*., 2009; Fujie *et al*., 2010), this approach has not been widely adopted for macro‐scale imaging. In practise, this is commonly due to limitations in sensitivity and difficulties in imaging acquisition and analysis.

An alternative approach is to use bacteria labelled with the *lux* operon. Bacterial bioluminescence (Huang *et al*., 2006; Rico *et al*., 2010; Brodl *et al*., 2018; Fleiss and Sarkisyan, 2019) offers several advantages over fluorescent proteins for macro‐scale imaging. Bioluminescence has generally little or no background, excitation light is not needed and emission of light depends on bacterial metabolism, so only active cells are visible (Gregor *et al*., 2018; Planas‐Marquès *et al*., 2020). Consequently, bacterial bioluminescence has become a valuable tool to monitor bacterial localization in plants (Fukui *et al*., 1996; Bogs *et al*., 1998; Seleem *et al*., 2008; Xu *et al*., 2010) and animals (Yu *et al*., 2004; Burkatovskaya *et al*., 2006; Mortin *et al*., 2007; Seleem *et al*., 2008) at a macroscopic scale. Advanced imaging technologies, such as electron multiplying CCD (EMCDD) cameras, can be used to detect bioluminescence at a single‐cell level, as primarily demonstrated in mammalian cell systems to date (Asai *et al*., 2008; Iwano *et al*., 2018). However, this equipment is not widely available within the scientific community. Therefore at present, bioluminescence is most often used for temporal analyses of cell growth, viability and gene expression in bacterial populations using luminometers and macroscopic imaging using standard CCD cameras.

In an attempt to solve the low signal intensity limitation, Gregor *et al*. (2018) recently developed an enhanced *lux* operon, called *ilux*, which could allow single‐cell imaging of *Escherichia coli*. The *ilux* operon consists of a series of random mutations within the *lux* operon and an *frp* gene from *Vibrio campbellii* inserted at the end of *luxE* to maximize brightness (Gregor *et al*., 2018). The *frp* gene codes for an FMN reductase responsible for generating FMNH_2_ which is then oxidized in the process of light production (Gregor *et al*., 2018). In *E. coli*, the additional FMN reductase caused a 2.3‐fold increase in brightness, suggesting that FMNH_2_ generated by the endogenous FMN reductase in *E. coli* was limiting for *lux* activity under the conditions tested (Gregor *et al*., 2018). However, this has not yet been tested in a wide range of bacteria and under a range of environmental conditions.

When using either fluorescent proteins or the *lux* operon as a bioreporter, the system used to tag bacteria has to be chosen carefully. Ideally, the marker genes should integrate into the bacterial chromosome in a single copy and in a neutral position to avoid disrupting endogenous gene functions (Lambertsen *et al*., 2004). These criteria are satisfied by the bacterial transposon Tn7 (Craig, 1991) which can be used to insert cloned DNA at high efficiency into a specific intergenic site *att*Tn7, present in the chromosome of many Proteobacteria (Koch *et al*., 2001).

This study aimed to develop a novel library of mini‐Tn7 delivery plasmids combining the benefits of both bacterial luminescence and fluorescence for macro‐ and micro‐scale resolution imaging, which can be used to chromosomally tag Proteobacteria. We assessed both *ilux* (Gregor *et al*., 2018) and modified *lux* operons (*luxCDABE* and *luxCDABE‐frp*, this study) in different bacterial strains to select the brightest constructs before developing the mini‐Tn7 vector library. We demonstrate that these constructs can be used to visualize both local and systemic host–microbe interactions between *Phaseolus vulgaris* and the bacterial plant pathogen *Pseudomonas syringae* pv. *phaseolicola* (*Pph*) and show that *Pph* can move systemically through bean leaves by colonizing the vascular system. While we have demonstrated the applicability of these constructs to study spatio‐temporal relationships in plant–microbe interactions, this toolbox will be instrumental to study a wide range of host–microbe, microbe–microbe and microbe–environment interactions *in situ*.

## Results and discussion

### Comparison between *lux*, *ilux* and *lux frp* operons

To select the brightest bioluminescence operon and achieve a low detection threshold when visualizing bacteria in the environment, we tested whether *ilux* (Gregor *et al*., 2018) was brighter than *lux* in bacteria other than *E. coli*. We developed a low copy‐number plasmid, called pRSJ‐p_nptII_::ilux (Supporting Information Table S1) derived from the reporter plasmid pIJ11282 (Frederix *et al*., 2014), in which *lux* was replaced with *ilux*. Additionally, to test the effect of *frp* on *lux‐*mediated bioluminescence, we included *frp* from pGEX(−) (Gregor *et al*., 2018) after *luxE* in pIJ11282, generating pRSJ‐p_nptII_::lux‐frp (Supporting Information Table S1). The three plasmids pIJ11282, pRSJ‐p_nptII_::ilux and pRSJ‐p_nptII_:lux‐frp have the same backbone and promoter.

pRSJ‐p_nptII_::ilux did not yield brighter bioluminescence than pIJ11282 in *Acinetobacter baylyi* strain ADP1, *Pseudomonas fluorescens* NZ011 and *Pseudomonas syringae* pv. *phaseolicola* (*Pph*) strain 1302A (Supporting Information Fig. S1A). On the contrary, in *P. fluorescens* NZ011, pRSJ‐p_nptII_::ilux was approximately 20‐fold less bright than pIJ11282 in both M9 minimal medium and King's B (KB) medium (Supporting Information Fig. 1A). We speculate that the mutations introduced into the *ilux* operon were optimized for light production under the conditions tested in *E. coli* and similar results are not guaranteed for other bacterial strains. However, pRSJ‐p_nptII_:lux‐frp was two‐fold brighter than pIJ11282 in *P. fluorescens* NZ011 without affecting bacterial growth (Supporting Information Fig. S1B), indicating that, depending on the bacterial strain, an additional FMN reductase can increase the amount of light produced. FMN reductases oxidize NADPH to NADP+ in order to reduce FMN to FMNH_2_. To test for the activity of the FMN reductase encoded by the *frp* gene, we measured the NADP+ concentration of cell extracts of *P. fluorescens* NZ011 harbouring pIJ11282 or pRSJ‐p_nptII_:lux‐frp. We observed a higher concentration of NADP+ in bacterial cells expressing the *frp* gene, which supports the conclusion that the increased bioluminescence of *P. fluorescens* NZ011 (pRSJ‐p_nptII_:lux‐frp) relative to *P. fluorescens* NZ011 (pIJ11282) is due to increased FMN reductase activity (Supporting InformationFig. S2).

### Generation of an improved mini‐Tn7 vector library for bioluminescence and combined bioluminescence‐fluorescence expression

To combine the benefit of using bacterial bioluminescence for macro‐scale localization with the advantages of fluorescent proteins for micro‐scale localization, we developed a library of mini‐Tn7 plasmids containing both a bioluminescent operon and eYFP (Supporting Information Fig. S3). eYFP was chosen over other fluorescent proteins for its higher brightness over eGFP (Shaner *et al*., 2005) and for its peak emission spectra (527 nm), which would easily allow visualization in an environment with red autofluorescence (*e.g*. leaves). To provide a wide range of expression levels of the bioluminescent operon, which would allow researchers to modularly choose the best expression level according to their experimental needs, we designed the library to have five different constitutive promoters driving *lux* transcription. Promoters were selected to be highly conserved, constitutive and to provide a range of expression levels. For these reasons, we chose the *recA* promoter (Giliberti *et al*., 2006), named *p*_*OXB20*_ and its derivatives *p*_OXB16_, *p*_OXB13_ and *p*_OXB11_ (Oxford Genetics Limited, https://www.oxgene.com/Products; Lynn *et al*., 2015; Presnell *et al*., 2019), and the promoter *p*_nptII_ (Wright and Beattie, 2004). We also included two different constitutive promoters driving the expression of eYFP, namely *p*_lac_ and *p*_A1/04/03_ (Koch *et al*., 2001). *p*_*lac*_ is a common and conserved constitutive promoter, while *p*_A1/04/03_ has been widely adopted for gene expression in *Pseudomonas* (Godfrey *et al*., 2010)..

To generate the mini‐Tn7 vector library, we used the two cassettes with the highest light production in the tested bacterial strains, which were the *lux* and the modified *lux* and *frp* operons (Supporting Information Fig. S1). Initially, we used pUC18‐mini‐Tn7T‐Gm‐lux (Choi *et al*., 2005) as a backbone. Plasmid pUC18‐mini‐Tn7T‐Gm‐lux was developed to easily insert a promoter of interest into a multiple cloning site (MCS) upstream of *luxC*. However, this backbone has a different ribosome binding site (RBS) upstream of *luxC* than the RBS found upstream of *luxC* in pIJ11282 (Frederix *et al*., 2014). The latter RBS has been predicted to increase the translation initiation rate more than fourfold (14243 arbitrary units compared to 3371 calculated with the RBS calculator; Farasat *et al*., 2014; Ng *et al*., 2015). Additionally, the upstream sequence of *luxC*, encompassing the MCS in pUC18‐mini‐Tn7T‐Gm‐lux was found to negatively affect transcription (Glassing and Lewis, 2015) (Supporting Information Fig. S4A). We therefore constructed a second plasmid (pRS‐p_OXB20_::lux‐p_A1/04/03_::eYFP) containing the RBS from pIJ11282 without the long MCS of pUC18‐mini‐Tn7T‐Gm‐lux (Supporting Information Fig. S4B).

We tested both constructs in *Pph* 1302A and found that the promoter with the alternate RBS and without the MCS of pUC18‐mini‐Tn7T‐Gm‐lux (*p*_OXB20_) resulted in four to seven times more luminescence compared to the original promoter (*p*_OXB20(1)_) in both KB and M9 media, with no consequences for bacterial growth (Supporting Information Fig. S5A and B).

**Fig. 1:**
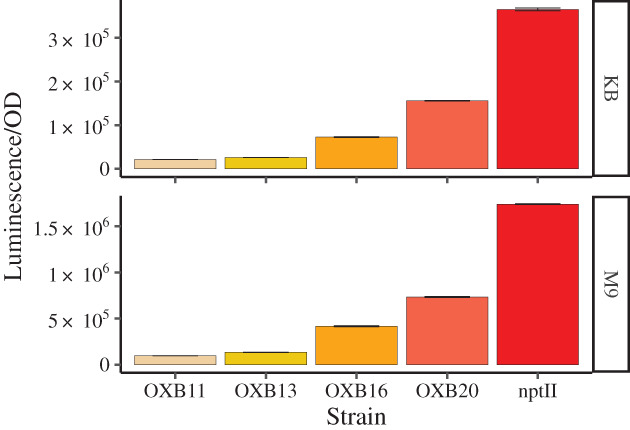
A library of mini‐Tn7 constructs with different promoters upstream of the*lux*operon provides a wide dynamic range of luminescence when introduced into*Pseudomonas syringae*pv*. phaseolicola*1302A (*Pph*1302A). Luminescence values were detected at 4 h. Time series data are presented in the Supporting Information Fig. S6.**OXB11**:*Pph*1302A p_OXB11_::lux‐p_A1/04/03_::eYFP;**OXB13**:*Pph*1302A p_OXB13_::lux‐p_A1/04/03_::eYFP;**OXB16**:*Pph*1302A p_OXB16_::lux‐p_A1/04/03_::eYFP;**OXB20**:*Pph*1302A p_OXB20_::lux‐p_A1/04/03_::eYFP;**nptII**:*Pph*1302A p_nptII_::lux‐p_A1/04/03_:::eYFP. KB (King's B medium), M9 (M9 minimal medium). OD = optical density at 600 nm. Error bar ± SE.*n* = 3. [Color figure can be viewed at wileyonlinelibrary.com]

As we found these backbones to be an improvement over constructs based on pUC18‐mini‐Tn7T‐Gm‐lux, we also included constructs with the *lux* operon by itself in our library, without eYFP (Table 1; Supporting Information Table S1). The five different constitutive promoters used encompass a 15‐fold range between the weakest promoter *p*_OXB11_ and the strongest promoter *p*_nptII_ in *Pph* 1302A (Fig. 1; Supporting Information Fig. S6A). We observed a small but significant decrease in bacterial growth both in *vitro* and *in planta* when comparing *Pph* 1302A with *Pph* 1302A p_nptII_::lux‐p_A1/04/03_::eYFP inoculated into the susceptible host plant *P. vulgaris* cultivar Canadian Wonder (Supporting InformationFig. 6B and Fig. 7A and B). However, we did not detect any difference between the growth of *Pph* 1302A and *Pph* 1302A p_OXB16_::lux‐p_A1/04/03_::eYFP (intermediate expression) or *Pph* 1302A p_OXB11_::lux‐p_A1/04/03_::eYFP (low expression) *in planta* (Supporting Information Fig. S7B).

**Table 1:** Mini‐Tn7 vector library constructed in this study.^a^

Lux	Lux and eYFP	Lux and eYFP
pRS‐p_nptII_::lux	pRS‐p_nptII_::lux‐p_A1/04/03_::eYFP	pRS‐p_nptII_::lux‐p_lac_::eYFP
pRS‐p_OXB20_::lux	pRS‐p_OXB20_::lux‐p_A1/04/03_::eYFP	pRS‐p_OXB20_::lux‐p_lac_::eYFP
pRS‐p_OXB16_::lux	pRS‐p_OXB16_::lux‐p_A1/04/03_::eYFP	pRS‐p_OXB16_::lux‐p_lac_::eYFP
pRS‐p_OXB13_::lux	pRS‐p_OXB13_::lux‐p_A1/04/03_::eYFP	pRS‐p_OXB13_::lux‐p_lac_::eYFP
pRS‐p_OXB11_::lux	pRS‐p_OXB11_::lux‐p_A1/04/03_::eYFP	pRS‐p_OXB11_::lux‐p_lac_::eYFP
pRS‐p_nptII_::lux‐frp	pRS‐p_nptII_::lux‐frp‐p_A1/04/03_::eYFP	pRS‐p_nptII_::lux‐frp‐p_lac_::eYFP
pRS‐p_OXB20_::lux‐frp	pRS‐p_OXB20_::lux‐frp‐p_A1/04/03_::eYFP	pRS‐p_OXB20_::lux‐frp‐p_lac_::eYFP

^a^ For more details, see the Supporting Information Table S1.

Access to a range of promoters will enable researchers to choose an expression level of the *lux* cassette that is suitable for their experimental conditions, balancing both signal strength and any fitness cost observed (*e.g*. purpose of the experiment, equipment used to detect luminescence). While we would expect the constructs to give different expression levels in different bacterial strains, the constructs should maintain a wide expression range as the OXB promoters (Oxford Genetics Limited, UK) were all derived from the constitutive and conserved *recA* promoter (Weisemann and Weinstock, 1991).

### Pseudomonas syringae pv. phaseolicola colonizes the vascular system of *Phaseolus vulgaris* leaves

We applied a combined luminescence‐fluorescence approach to investigate whether *Pph* systemically infects *P. vulgaris* leaves. *Pph*, the causal agent of halo blight of bean, is known to be a seed‐borne pathogen and to be able to cause systemic symptoms and spread within infected plants (Taylor *et al*., 1979), but to date, there is limited knowledge of the routes and mechanisms used by *Pph* to spread systemically within host plants.

We tagged *Pph* RJ3 (Jackson *et al*., 2000) using pRS p_nptII_::lux‐p_A1/04/03_::eYFP (Table 1, Supporting Information Table S1) as *p*_nptII_ was the strongest promoter (Fig. 1) and p_A1/04/03_ has previously been successfully used in *Pph* (Godfrey *et al*., 2010)*. Pph* RJ3 differs from *Pph* 1302A as it lacks a > 40 Kb genomic island that contains the gene *avrPphB*, which encodes a secreted effector protein that elicits effector triggered immunity (ETI) in plants carrying the resistance gene *R3* (Jackson *et al*., 2000). Due to the loss of *avrPphB*, *Pph* RJ3 can successfully colonize *P. vulgaris* cultivar Tendergreen (TG) without triggering a plant immune response known as the hypersensitive response (HR), which acts to restrict bacterial growth. In experiments with bacteria incubated in agar plates, we calculated that the detection limit for tagged cells, in terms of number of cells detectable per unit area, was approximately 100 CFU mm^−2^ (Supporting Information Fig. S8) and observed a high correlation (0.99) between luminescence and CFU, as also reported by Fan *et al*. (2008).

**Fig. 2:**
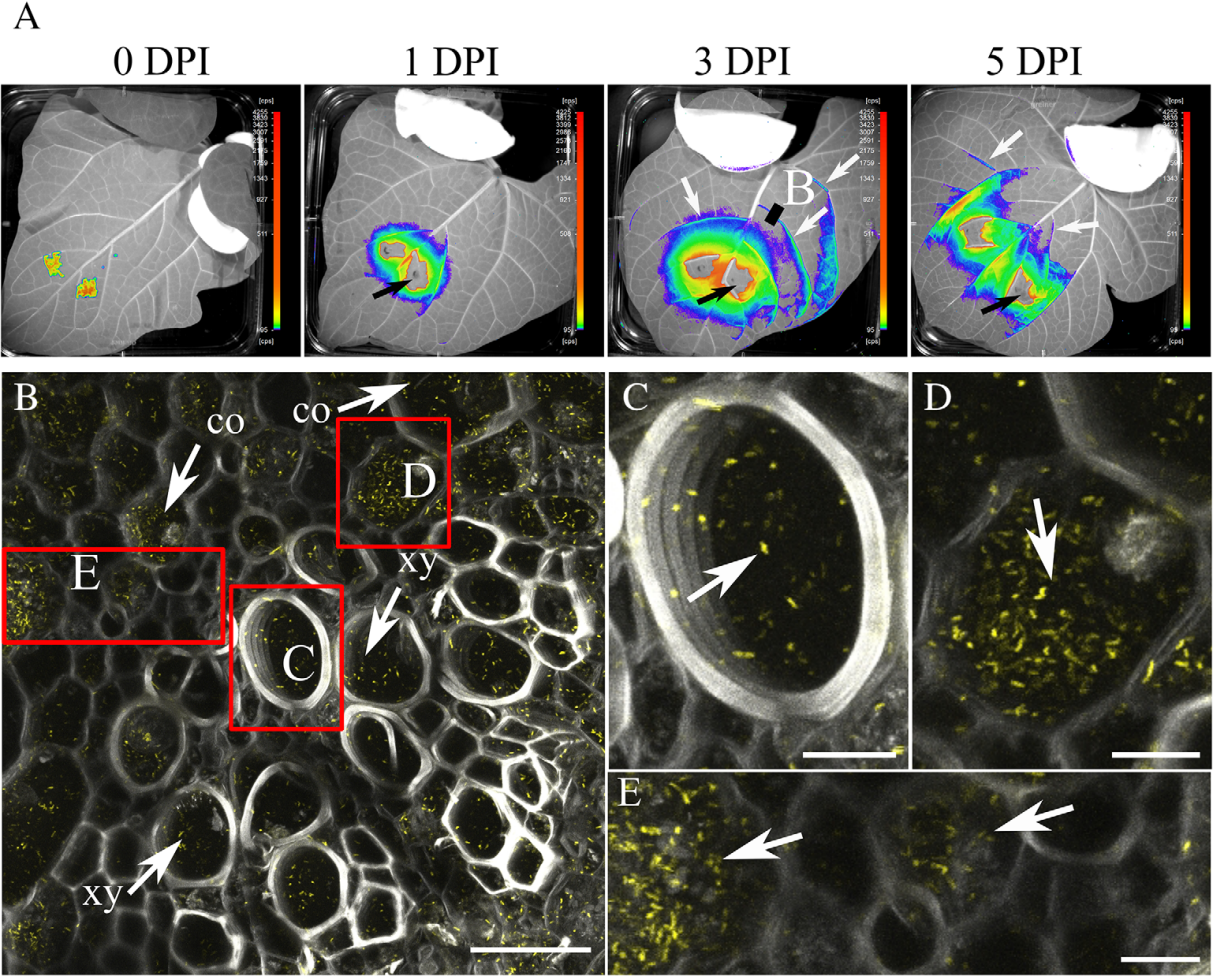
*Pseudomonas syringae* pv. *phaseolicola* RJ3 (*Pph* RJ3) colonizes the leaf vasculature of *P. vulgaris* cultivar TG. A. *Pph* RJ3 p_nptII_::lux‐p_A1/04/03_::eYFP was syringe‐infiltrated in localized areas on the abaxial surface of *Phaseolus vulgaris* cv. Tendergreen leaves. Images were taken after 0, 1, 3, 5 DPI with the nightOWL LB 983 at 10‐min exposure. The black line indicates cross sections used for confocal microscopy imaging (B). The white arrows indicate co‐localization of *Pph* RJ3 p_nptII_::lux‐p_A1/04/03_::eYFP with the leaf vasculature. The black arrows indicate regions of the leaves that have cts values higher than the maximum detection limit. B. Maximum projections of the leaf vasculature cross section. *Pph* RJ3 p_nptII_::lux‐p_A1/04/03_::eYFP colonies (yellow). Xylem autofluorescence (bright grey). Collenchyma autofluorescence (dark grey). co; collenchyma. xy; xylem. Red squares indicate areas shown at higher magnification in panels C–E. Scale bar: 40 μm. C. *Pph* RJ3 p_nptII_::lux‐p_A1/04/03_::eYFP colonies (yellow; white arrow). Xylem autofluorescence (grey). D. *Pph* RJ3 p_nptII_::lux‐p_A1/04/03_::eYFP colonies (yellow; white arrow). Collenchyma autofluorescence (dark grey). E. Zoom of panel B. *Pph* RJ3 p_nptII_::lux‐p_A1/04/03_::eYFP colonies (yellow; white arrow). Collenchyma autofluorescence (dark grey). Scale bar: 10 μm. [Color figure can be viewed at wileyonlinelibrary.com]

We used bacterial bioluminescence to macroscopically observe *Pph* RJ3 in *P. vulgaris* cultivar TG leaves. We detected a clear co‐localization of *Pph* RJ3 with the leaf vasculature within 3 days after syringe infiltration (Fig. 2A). We then used this information to select and collect leaf samples (vasculature cross sections) from leaves infected with *Pph* RJ3 to be visualized with confocal microscopy. This confirmed that *Pph* RJ3 was localized inside the leaf vasculature (Fig. 2B and C). Both data types (bioluminescence and fluorescence) showed the ability of *Pph* RJ3 to move from the infection site.

We were able to detect *Pph* RJ3 cells within the xylem vessels (Fig. 2B and C), and in the collenchyma (Fig. 2B, D, and E). *Pph* RJ3 appeared to accumulate to higher densities in the collenchymatic cells of the leaf vascular system than in the xylem (Fig. 2D and E; Supporting Information Video S1). *Pph* RJ3 movement in the xylem was more rapid than movement observed in collenchymatic cells (Supporting Information Video S2), suggesting that *Pph* RJ3 moves within, but does not produce biofilms inside the xylem. We confirmed that *Pph* RJ3 can colonize the vascular system of intact leaves following epiphytic colonization by spray‐inoculating *Pph* RJ3 onto *P. vulgaris* leaves (Supporting Information Fig. S9). Images of negative controls show that there is little or no background signal in the absence of tagged bacteria (Supporting Information Fig. S10).

*Pph* strains have been reported to show substantial variation in their ability to cause systemic symptoms in host plants, while host cultivars have been reported to vary in their susceptibility to systemic symptoms. This has been attributed in some studies to systemic movement of the toxin, phaseolotoxin, within host plants (Mitchell and Bieleski, 1977). Our study confirms that the pathogen itself can move systemically within host tissues, although under our experimental conditions *Pph* RJ3 was not an aggressive systemic pathogen, consistent with the absence of detectable bacterial biofilms in the xylem and wilting symptoms, which are often the manifestation of systemic bacterial infections (Bae *et al*., 2015). The constructs and methods established in this study will provide valuable tools to investigate the underlying causes of variation in systemic infection and will enable researchers to better understand the biology and epidemiology of this disease.

### Bacterial bioluminescence can be used to monitor the effect of plant immune responses on bacterial growth and viability *in planta*

Bacterial bioluminescence has been proposed as a high‐throughput method to detect *in planta* growth of bacterial pathogens (Fan *et al*., 2008). Fan *et al*. (2008) showed that *Pseudomonas syringae* tagged with *luxCDABE* can be used as a quantitative assay to monitor bacterial growth and that CFU is correlated with bacterial bioluminescence. Moreover, luminescence allows the detection of bacterial growth without performing tissue extraction, serial dilutions, plating, and scoring, thus reducing the time needed for the experiment. We therefore examined whether the luminescent constructs developed in this study could also be used to rapidly and sensitively monitor the effect of different plant immune responses on bacterial growth and viability *in planta*. As expression of high levels of *lux* activity could have a detrimental effect on bacterial fitness in the plant environment, we examined whether a relatively weak promoter driving *lux* expression could be used for this purpose.

We tagged *Pph* 1302A Δ*hrpA* (Supporting Information Table S2), *Pph* 1302A Δ*xerC* (Lovell *et al*., 2009) and *Pph* 1302A Δ*avrPphB* (Supporting Information Table S2) using pRS‐p_OXB13_::lux (Table 1; Supporting Information Table S1). *Pph* 1302A Δ*xerC* (Lovell *et al*., 2009) lacks a recombinase (XerC) that is able to excise the genomic island containing *avrPphB*, thus it stably triggers ETI in *P. vulgaris* cultivar TG. On the contrary, *Pph* 1302A is able to excise the island and overcome resistance. *Pph* 1302A Δ*hrpA* (this study; Supporting Information Table S2) cannot assemble a functioning type III secretion system (T3SS), thus it cannot secrete effectors to counteract plant defences. Therefore, its growth *in planta* is suppressed by PAMP‐Triggered Immunity (PTI) (Jones and Dangl, 2006). *Pph* 1302A Δ*PphB* (this study; Supporting Information Table S2) has a functional T3SS, but lacks the *avr* gene *avrPphB*, thus it can suppress PTI and colonize TG leaves. This compatible interaction is named effector‐triggered susceptibility (ETS) (Jones and Dangl, 2006).

**Fig. 3:**
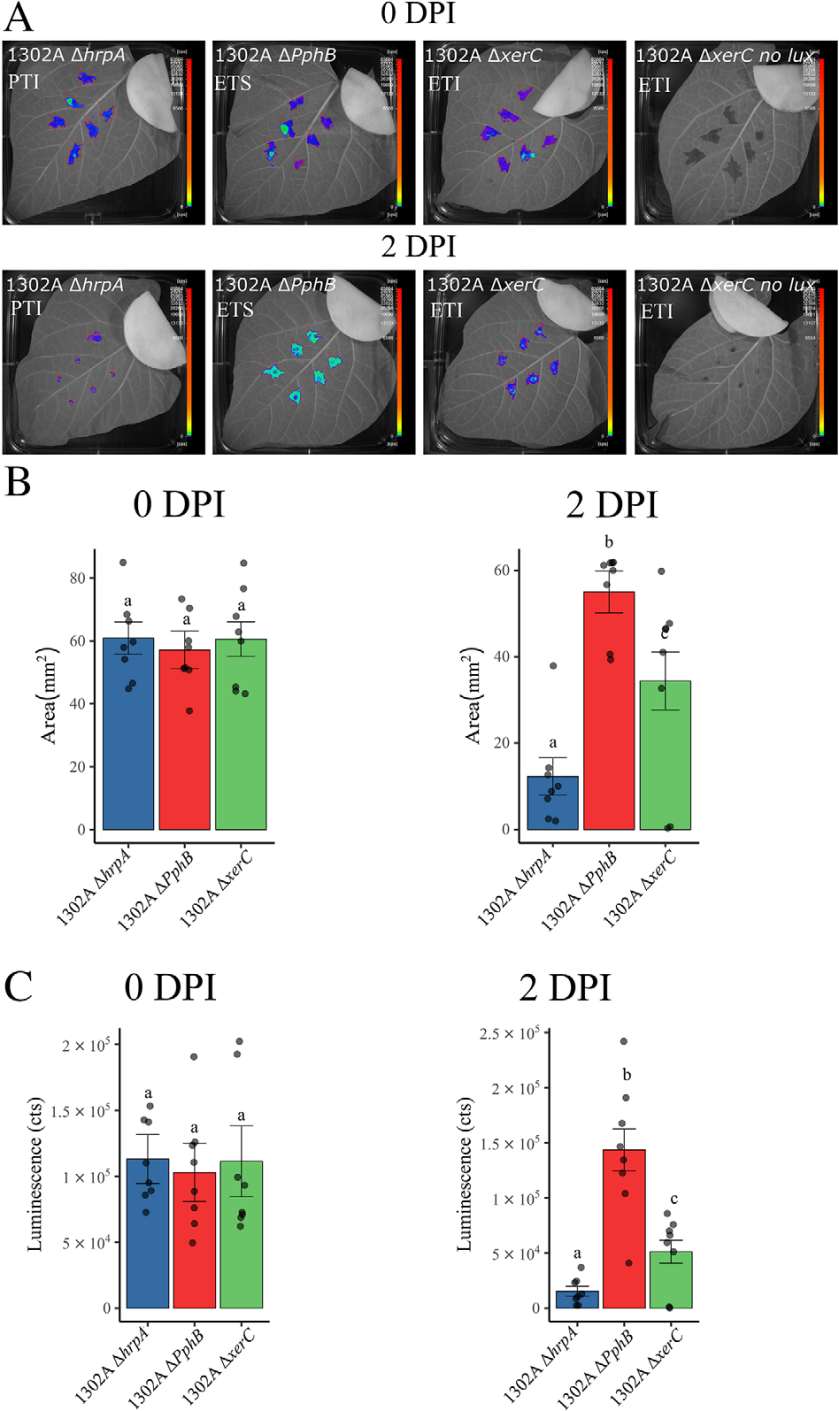
Bacterial bioluminescence can be used to study the effect of plant immune responses on bacterial growth and viability*. Phaseolus vulgaris* cultivar Tendergreen leaves were syringe infiltrated with 10^6^ CFU ml^−1^ of *Pseudomonas syringae* pv*. phaseolicola* 1302A (*Pph* 1302A) Δ*hrpA*, Δ*PphB* and Δ*xerC* tagged with p_OXB13_::lux. A. After infiltration, leaves were kept in the dark for 10 min and then imaged with the nightOWL LB 983 at 2 min exposure. B. After 2 days post inoculation, leaves were detached from the plants, kept in the dark for 10 min and imaged with the nightOWL LB 983 at 5 min exposure. **1302A Δ*PphB***: Pph 1302A Δ*PphB* p_OXB13_::lux; **1302A Δ*xerC***: Pph1302A Δ*xerC* p_OXB13_::lux; **1302A Δ*hrpA***: Pph 1302A Δ*hrpA* p_OXB13_::lux; **1302A Δ*xerC* no lux**: Pph 1302A Δ*xerC*. Luminescence and area values are reported for the eight biological replicates. Technical replicates (spots within the same leaf) were averaged. Error bar ± SE. *n* = 8. Significant differences (Student's T‐test, *P* < 0.05) are indicated by letters. [Color figure can be viewed at wileyonlinelibrary.com]

A 0 days post inoculation (DPI) we could not detect any difference between strains for both luminescence value (cts) [ANOVA, *F*(2,14) = 0.0616,*P* = 0.94] and infected area (mm^2^) [ANOVA, *F*(2,14) = 0.18, *P* = 0.83] (Fig. 3A–C). At 2 DPI, both luminescence signal (cts) [ANOVA, *F*(2,14) = 35.01, *P* < 0.0001] and infected area (mm^2^) [ANOVA, *F*(2,14) = 19.21, *P* < 0.0001] for the different strains displayed significant differences using a two way ANOVA, as well as pairwise comparisons between strains (Fig. 3A–C), indicating that both bacterial bioluminescence and infected area can be used to detect the effect of different plant immune responses on bacterial viability and growth, with significantly lower bioluminescence in the context of PTI and ETI.

Colony count analyses performed at 2 DPI agreed with luminescence data [ANOVA, *F*(2,14) = 56.73, *P* < 0.0001] identifying a statistically significant effect of strain. However, pairwise comparisons failed to distinguish between plants infected with *Pph* 1302A Δ*xerC* and *Pph* 1302A Δ*PphB* [t(14) = 1.6, *P* = 0.06] (Supporting Information Fig. 11A and B). The higher variability of colony count data compared to bioluminescence data (Fig. 3A–C; Supporting Information Fig. S11B) may be associated with variability introduced during tissue extraction, serial dilution and plating that are avoided in bioluminescence detection. Therefore, the bioluminescent reporter constructs developed in this study could be used to effectively monitor the effect of plant immune responses on bacterial growth, spread and viability in interactions between *Pph* and *P. vulgaris*.

## Conclusion

Dual bioluminescence and fluorescence reporter constructs have previously been described for both gram‐negative and gram‐positive bacteria and used to study transcription, metabolic activity and host colonization (Unge *et al*., 1999; Unge and Jansson, 2001; Perehinec *et al*., 2007; Benedetti *et al*., 2012; Kim *et al*., 2018). Here, we describe the development of a flexible library of mini‐Tn7 constructs that can be used to tag Gram‐negative bacteria with a combination of *lux*, *lux‐frp* and *lux‐*eYFP operons with a wide selection of promoter strengths to drive *lux* expression. The strongest promoter used (*p*_*nptII*_), together with a modified RBS and removal of the MCS, gives a fourfold to sevenfold increase in luminescence over a previously described mini‐Tn7‐*lux* construct. We also show that introducing an additional FMN reductase can positively increase bioluminescence in some bacterial strains and that the relative performance of *lux* and *ilux* depends on the bacterial strain used.

We have demonstrated the application of these reporter constructs by showing that *Pph* is able to systemically colonize the leaves of *P. vulgaris*. By combining bioluminescence and fluorescence, the process of collecting samples to be imaged with confocal microscopy could be greatly accelerated. Interestingly, *Pph* did not form biofilms within the xylem vessels, where they were observed to be highly mobile. Instead *Pph* preferentially colonized collenchymatic cells. While previous studies have shown that *Pph* causes systemic symptoms in *P. vulgaris* (Zaiter and Coyne, 1984), the question of whether, and how *Pph* is able to move systemically through host plants has not been fully addressed. Having shown that *Pph* can colonize the leaf vascular system it will be of interest to elucidate the molecular mechanisms underpinning vascular colonization by *Pph* and to investigate the contribution of vascular colonization to the development of systemic symptoms and to seed‐borne transmission of *Pph*.

Our results also show that bacterial bioluminescence can be used to rapidly detect the effect of different plant immune responses on bacterial viability and growth and to monitor the effectiveness of plant immune responses in restricting systemic colonization of plant tissues. Importantly, bioluminescence is a sensitive indicator of bacterial viability and metabolic activity as well as localisation, while the relatively high stability of fluorescent proteins means that they can be used to detect both living and metabolically inactive or dead cells. Thus, it is of interest to note that while ETI and PTI could be observed to restrict the movement and population levels of *Pph* in infected tissue, we were still able to detect a substantial number of bioluminescent cells 2 days, and even 20 days after infection (Fig. 3A; data not shown), showing that neither PTI nor ETI eliminated invading bacteria. Bioluminescent imaging may be of particular value in studying diseases such as bacterial leaf streak of wheat caused by *Xanthomonas translucens* pv. *undulosa*, or bacterial blight of rice caused by *Xanthomonas oryzae* pv. *oryzae*, in which resistance is typically assessed by monitoring lesion length or area, allowing rapid comparative imaging of bacterial spread within host plants even before lesions develop (Kandel *et al*., 2012; Oliva *et al*., 2019; Sapkota *et al*., 2020). We have already confirmed that the constructs described here can be used to transform and produce bioluminescence in *Xanthomonas* (data not shown).

Mini‐Tn7 constructs have previously been used to transform a wide range of Proteobacteria by exploiting the presence of a conserved *att*Tn7 site downstream of the *glmS* gene, in which the effects of transposon insertion have generally been found to be relatively neutral (Choi *et al*., 2005; Choi and Schweizer, 2006). More recently, researchers have shown that the range of bacteria that can be tagged using this system can be further expanded through the introduction of an artificial *att*Tn7 site, which can also be used to extend the use of this system to bacteria in which insertion into the pre‐existing *att*Tn7 site has been found to not be neutral (Figueroa‐Cuilan *et al*., 2016). We therefore believe that the constructs and approaches described in this study will be a valuable resource for researchers who are interested in tracking both the spatio‐temporal and physiological dynamics of bacterial populations in a wide range of biological contexts.

## Experimental procedures

### Bacterial strains, media and growth conditions

*E. coli* DH5α strains carrying the plasmids listed in the Supporting Information Table S1 were inoculated from frozen glycerol stock onto Luria Bertani (LB) (Sambrook *et al*., 1989) agar (1.5% agar) and grown for 24 h at 37°C. Single colonies were used to inoculate 10 ml LB cultures, which were incubated at 37°C with shaking at 200 r.p.m. *Pseudomonas* and *Acinetobacter* strains were routinely grown in the same manner but incubated at 28°C. Antibiotics were included where relevant at the following concentrations: gentamicin (10 μg ml^−1^), tetracycline (10 μg ml^−1^), kanamycin (20 μg ml^−1^), carbenicillin (50 μg ml^−1^), nitrofurantoin (25 μg ml^−1^).

### Plant growth conditions

*Phaseolus vulgaris* cultivars Tendergreen (TG) and Canadian Wonder were grown at 20–22°C, 70% humidity and with artificial light maintained for 8 h periods within the 24‐h cycle. Two‐week old plants (first true leaves fully developed) were used for all experiments.

### Cloning, restriction enzyme digestion and polymerase chain reaction

Cloning was performed using Gibson assembly (Gibson *et al*., 2009) and constructs heat‐shock transformed into *E. coli* strain DH5α. Transformants harbouring the *lux* operon were screened for luminescence using a nightOWL LB 983 *in vivo* imaging system (Berthold Technologies, Germany). For constructs combining luminescence and fluorescence, transformants were screened for both luminescence and fluorescence using a nightOWL LB 983 *in vivo* imaging system (Berthold Technologies) and a FastGene blue light LED illuminator (Geneflow Ltd, UK) respectively. PCR was conducted according to the manufacturer's instructions using Q5 high‐fidelity DNA polymerase (Thermo Fisher Scientific) unless otherwise specified. Colony‐PCR was performed according to the manufacturer's instructions using GoTaq Green Master Mix (Promega Corporation, USA). DNA Sanger sequencing was performed by Source Bioscience (UK). Restriction enzyme digestions were performed according to the manufacturer's instructions (Thermo Fisher Scientific).

### Generation of a low copy‐number plasmid harbouring *ilux* and *lux* constructs

pRSJ‐p_nptII_::ilux was derived from pIJ11282 (Frederix *et al*., 2014) and pGEX(−) (Gregor *et al*., 2018). Briefly, pIJ11282 was digested with PstI and SnaBI to remove the *lux* operon. *ilux* was amplified with primers OXRS_F1 and OXRS_R1 (Supporting Information Table S3) from pGEX(−). The PCR product was ligated into PstI/SnaBI‐digested pIJ11282 and transformed into *E. coli*. Potential clones were selected in LB agar supplemented with 10 μg ml^−1^ tetracycline. DNA sequencing was performed with primers listed in the Supporting Information Table S3.

pRSJ‐p_nptII_::lux‐frp was generated by ligating *frp* from pGEX(−) into pIJ11282. Briefly, *frp* was amplified with primer OXRS_F2 and OXRS_R2 (Supporting Information Table S3) from pGEX(−) and ligated into PstI‐digested pIJ11282. Transformants were selected on LB agar supplemented with 10 μg ml^−1^ tetracycline. DNA sequencing was performed using primer OXRS_S1 (Supporting Information Table S5).

### Generation of a mini‐Tn7 vector library for *lux* expression

pRS‐p_OXB20(1)_::lux was generated by ligating the OXB20 promoter (Oxford Genetics Limited, UK) synthesized by Eurofins Genomics into SmaI‐digested pUC18‐mini‐Tn7T‐Gm‐lux (Choi *et al*., 2005). To make pUC18‐mini‐Tn7T‐Gm‐lux compatible for conjugation, oriT was PCR amplified with primers OXRS_FT and OXRS_RT (Supporting Information Table S3) from pK18mobsacB (Schäfer *et al*., 1994) and ligated into EheI‐digested pUC18‐mini‐Tn7T‐Gm‐lux. Potential clones were selected on LB agar supplemented with 10 μg ml^−1^ gentamicin. DNA sequencing was performed using primer OX26 (Supporting Informationy Table S4).

pRS‐p_nptII_::lux was generated by ligating *lux* amplified with primers OXRS_F3 and OXRS_R3 (Supporting Information Table S3) from pIJ11282 into the pUC18T backbone amplified with primers OXRS_F4 and OXRS_R4 (Supporting Information Table S3) from pUC18T‐mini‐Tn7T‐Gm‐dsRedExpress (Choi *et al*., 2005). Transformants were selected on LB agar supplemented with 10 μg ml^−1^ gentamicin. DNA sequencing was performed using primers listed in the Supporting Information Table S6.

pRS‐p_OXB20_::lux, pRS‐p_OXB16_::lux, pRS‐p_OXB13_::lux and pRS‐p_OXB11_::lux plasmids were generated by ligating OXB20, OXB16, OXB13 and OXB11 promoters (Oxford Genetics Limited, UK) into PdiI (NaeI)/SnaBI‐digested pRS‐p_nptII_::lux. Transformants were selected on LB agar supplemented with 10 μg ml^−1^ gentamicin. DNA sequencing was performed using primer OXB26 (Supporting Information Table S4).

For constructs pRS‐p_OXB20_::lux and pRS‐p_nptII_::lux, we additionally included *frp* downstream of the *lux* operon. Briefly, *frp* was amplified from pGEX(−) with primers OXRS_F5 and OXRS_R5 (Supporting Information Table S3) and ligated into MscI‐digested pRS‐p_nptII_::lux and MscI‐digested pRS‐p_OXB20_::lux. DNA sequencing was performed using primer OXRS_S1 (Supporting Information Table S5).

### Generation of a mini‐Tn7 vector library for combined bioluminescence and fluorescence expression

p_A1/04/03_::eYFP amplified with OXRS_F6 and OXRS_R6 (Supporting Information Table S3) from miniTn7(Gm)PA1/04/03‐eyfp‐a (Klausen *et al*., 2003), was ligated into MscI‐digested pRS‐p_nptII_::lux, pRS‐p_OXB20_::lux, pRS‐p_OXB16_::lux, pRS‐p_OXB13_::lux and pRS‐p_OXB11_::lux plasmids. p_A1/04/03_::eYFP amplified with OXRS_F7 and OXRS_R7 (Supporting Information Table S3) from miniTn7(Gm)PA1/04/03‐eyfp‐a (Klausen *et al*., 2003), was ligated into MscI‐digested pRS‐p_nptII_::lux‐frp and pRS‐p_OXB20_::lux‐frp. Transformants were selected on LB agar supplemented with 10 μg ml^−1^ gentamicin. For plasmids pRS‐p_nptII_::lux‐p_A1/04/03_::eYFP, pRS‐p_OXB20_::lux‐p_A1/04/03_::eYFP, pRS‐p_OXB16_::lux‐p_A1/04/03_::eYFP, pRS‐p_OXB13_::lux‐p_A1/04/03_::eYFP and pRS‐p_OXB11_::lux‐p_A1/04/03_::eYFP, DNA sequencing was performed using primer OXRS_S1 (Supporting Information Table S5). For plasmids pRS‐p_nptII_::lux‐frp‐p_A1/04/03_::eYFP and pRS‐p_OXB20_::lux‐frp‐p_A1/04/03_::eYFP DNA sequencing was performed using primer OXRS_S2 (Supporting Information Table S5).

The same cloning procedure, primers and PCR conditions used to ligate p_A1/04/03_::eYFP into pRS‐p_nptII_::lux, pRS‐p_OXB20_::lux, pRS‐p_OXB16_::lux, pRS‐p_OXB13_::lux, pRS‐p_OXB11_::lux, pRS‐p_nptII_::lux‐frp and pRS‐p_OXB20_::lux‐frp plasmids were used to amplify p_lac_::eYFP from pRS p_lac_::eYFP (this study) and ligate it into MscI‐digested pRS‐p_nptII_::lux, pRS‐p_OXB20_::lux, pRS‐p_OXB16_::lux, pRS‐p_OXB13_::lux, pRS‐p_OXB11_::lux, pRS‐p_nptII_::lux‐frp and pRS‐p_OXB20_::lux‐frp.

pRS‐p_OXB20(1)_::lux‐p_A1/04/03_::eYFP was generated by ligating p_A1/04/03_::eYFP amplified with primers OXRS_F8 and OXRS_R8 from miniTn7(Gm)PA1/04/03‐eyfp‐a (Klausen *et al*., 2003) into StuI‐digested pRS‐p_OXB20(1)_::lux. Potential clones were selected on LB agar supplemented with 10 μg ml^−1^ gentamicin.

### Construction of *Pph* 1302A *PphB* and *hrpA* knock‐out mutants

To generate the *Pph* 1302A *avrPphB* knock‐out mutant (*Pph* 1302A *PphB‐*), 1 kb genomic regions flanking *PphB* were PCR amplified with primers OXRS_F9 and OXRS_R9 (left end) and primers OXRS_F10 and OXRS_R10 (right end) (Supporting Information Table S3). Primer OXRS_R9 was designed to include the starting codon of *avrPphB* while primer OXRS_R10 included the stop codon of *avrPphB*. PCR products were ligated into pK18mobsacB (Schäfer *et al*., 1994) amplified with primers OXRS_F11 and OXRS_R11 (Supporting Information Table S3). Transformants were selected in LB agar supplemented with 20 μg ml^−1^ kanamycin. Colony‐PCR was performed with primers OXRS_F12 and OXRS_R12 (Supporting Information Table S3) to validate the assembly. The plasmid was conjugated into *Pph* 1302A, as described below, and transformants were plated onto LB plates supplemented with kanamycin and nitrofurantoin. Transformants were the result of plasmid integration (a single recombination event). To allow allelic exchange, four colonies were cultured overnight in LB and 20 μl plated onto LB (1.5% agar) supplemented with 10% sucrose. Colony PCR with primers OXRS_F9 and OXRS_R10 (Supporting Information Table S3) was used to confirm the deletion of *avrPphB* from the *Pph* 1302A genome.

A similar procedure was used to generate *Pph* 1302A *ΔhrpA*. Briefly, *hrpA* upstream and downstream regions were amplified from the *Pph* 1302A genome with primers OXRS_F13, OXRS_R13 and OXRS_F14, OXRS_R14 (Supporting Information Table S3) and ligated into pK18mobsacB amplified with primers OXRS_F11 and OXRS_R11 (Supporting Information Table S3). Conjugation and selections of knock‐out mutants were carried out as described for *Pph* 1302A Δ*PphB*.

### Mini‐Tn7 delivery by four‐parental mating conjugation

*Pph* 1302A strains were tagged by four‐parental mating conjugation (Choi and Schweizer, 2006) with minor modifications. Details of the protocol used can be found in the Supporting Information File S1. *Pph* 1302A transformants were screened for luminescence (if tagged with *lux* constructs) and luminescence and fluorescence (if tagged with *lux* and eYFP constructs) as reported for *E. coli*. The correct insertion of the mini‐Tn7 delivery construct was verified by colony‐PCR using primers OXRS_F15 and OXRS_R15 (Supporting Information Table S3). Primer OXRS_F15 anneals in the *glmS* gene of *Pph* and primer OXRS_R15 binds in the Tn7R (right end of the Tn7 transposon).

### Three‐parental mating conjugation

*Pseudomonas* and *Acinetobacter* strains (Supporting Information Table S2) were inoculated from frozen glycerol stock onto Luria Bertani (LB) (Sambrook *et al*., 1989) agar (1.5% agar) and grown for 24 h at 28°C. Single colonies were used to inoculate 10 ml LB cultures, which were incubated at 28°C with shaking at 200 rpm. Bacterial cultures were then used for three‐parental mating conjugation as described for four‐parental mating conjugation (Supporting Information File S1), using the helper plasmid pRK2013 (Knauf and Nester, 1982).

### Plate reader assay

Bacterial cultures were inoculated from frozen glycerol stock onto Luria Bertani (LB) (Sambrook *et al*., 1989) agar (1.5% agar) and grown for 24 h at 28°C. Single colonies were used to inoculate 10 ml LB cultures, which were incubated at 28°C with shaking at 200 rpm. After 24 h, 500 μl pre‐culture was used to set up an overnight 10 ml LB culture. Cultures were centrifuged at 4000*g* for 6 min and resuspended in 10 ml 10 mM MgCl_2._ The 15 μl of resuspended bacterial cultures were added to 135 μl King's B medium (KB) (King *et al*., 1954) or M9 medium (20% glucose) (CSH protocols) placed into a 96‐well assay black plate (Corning incorporated, USA). Luminescence and OD_600_ were measured using a Infinite M200 plate reader (Tecan Group Ltd, Switzerland) over 20 h. The temperature was set at 28°C and measurements were taken every 30 min.

### NADP+ assay

Bacterial cultures were inoculated from frozen glycerol stock onto Luria Bertani (LB) (Sambrook *et al*., 1989) agar (1.5% agar) supplemented with 10 μg ml^−1^ tetracycline and grown for 24 h at 28°C. Single colonies were used to inoculate 10 ml LB cultures supplemented with 10 μg ml^−1^ tetracycline, which were incubated at 28°C with shaking at 200 rpm. After 24 h, 200 μl pre‐culture was used to set up 200 ml LB cultures supplemented with 10 μg ml^−1^ tetracycline which were incubated at 28°C with shaking at 200 rpm for 6 h. Cultures were processed according to the manufacturer's instructions (NADP/NADPH assay ab176724, Abcam, UK) at a final OD_600_ of 3,3.

### Determining the effect of bacterial bioluminescence expression on bacterial growth *in planta*

*Pph* strains were inoculated from frozen glycerol stock onto Luria Bertani (LB) (Sambrook *et al*., 1989) agar (1.5% agar) and grown for 24 h at 28°C. Single colonies were used to inoculate 10 ml LB cultures, which were incubated at 28°C with shaking at 200 rpm. After 24 h, 500 μl pre‐culture was used to set up an overnight 10 ml LB culture. Cultures were centrifuged at 4000*g* for 6 min and resuspended in 10 ml 10 mM MgCl_2_ (5 × 10^6^ CFU ml^−1^). Four plants per bacterial culture were used for the experiment. Briefly, the first two fully developed leaves of 12 two‐weeks old plants (*P. vulgaris* cultivar Canadian Wonder) were syringe‐infiltrated with *Pph 1302A* p_nptII_::lux p_A1/04/03_, *Pph 1302A* p_OXB16_::lux p_A1/04/03_, *Pph 1302A* p_OXB11_::lux p_A1/04/03_ and *Pph 1302A* (5 × 10^6^ CFU ml^−1^) (Supporting Information Table S2). Each leaf was infiltrated four times in four small areas (50–80 mm^2^). The four spots within the same leaf were considered technical replicates and were averaged for the calculation of the CFU mm^−2^. At 2 DPI (Days Post Inoculation) four leaf discs per leaf (four leaves total per bacterial culture) were detached from the four inoculated spots with a 1 cm^2^ cork and pooled together. Samples were ground with a TissueLyser (Qiagen) in 1 ml 10 mM MgCl_2_, and serial dilutions (20 μl drops) were spotted onto LB agar supplemented with nitrofurantoin 25 μg ml^−1^. Plates were incubated at 28°C for 3 days. At 5 DPI, the same process described above was repeated.

### Determining the detection limit of *Pph* RJ3 p_nptII_ ::lux p_A1_ _/04/03_::eYFP

Bacterial cultures were prepared as described above. Serial dilutions (100/1000 μl) were performed in 10 mM MgCl_2_ and 100 μl aliquots were spotted onto LB (1.5% agar) square plates. Plates were incubated at 28°C for 1 h before imaging with the nightOWL LB 983 *in vivo* imaging system (Berthold Technologies) at 20 min exposure. Luminescence values and area (mm^2^) of each spot were measured for the 10^−1^, 10^−2^, 10^−3^, and 10^−4^ dilutions. Plates were then incubated at 28°C for 3 days to allow bacterial growth and colony count to be performed.

### Combined luminescence‐fluorescence approach to monitor *Pph* dispersal *in planta*

#### Syringe‐infiltration

*Pph* RJ3 p_nptII_::lux p_A1/04/03_::eYFP (Supporting Information Table S1) was inoculated from frozen glycerol stock onto Luria Bertani (LB) (Sambrook *et al*., 1989) agar (1.5% agar) and grown for 24 h at 28°C. Single colonies were used to inoculate 10 ml LB cultures, which were incubated at 28°C with shaking at 200 rpm. After 24 h, 500 μl pre‐culture was used to set up an overnight 10 ml LB culture. Cultures were centrifuged at 4000*g* for 6 min and resuspended in 20 ml 10 mM MgCl_2_. The bacterial suspension (5 × 10^6^ CFU ml^−1^) was syringe‐infiltrated into abaxial areas of the first true leaves of TG plants without causing any damage to the leaf mesophyll. To minimize damage to the leaf while infiltrating, we modified a 10 ml syringe by attaching to it a P20 pipette tip. We removed the last 1 cm of the P20 pipette tip and attached to it a rubber O‐ring to ensure a soft contact between the tip and the leaf mesophyll. Plants were incubated for 0,1,3 and 5 days at 22°C and artificial light was maintained for 16 h within the 24‐h cycle.

At designated times (0, 1, 3, and 5 DPI), leaves were detached and placed on square Petri dishes. Leaf petioles were wrapped in water‐soaked cotton discs to allow plant transpiration. Leaves were incubated in the dark for 10 min before imaging with the nightOWL LB 983 *in vivo* imaging system (Berthold Technologies) at 10 min exposure. Bacterial bioluminescence was used to visualize *Pph* localisation and to identify portions of leaves to be imaged with confocal laser scanning microscopy. Leaf vasculatures showing *Pph* colonization were cross‐sectioned by hand‐sectioning (50–80 μm) without embedding. Samples were imaged with a confocal laser scanning microscope Zeiss LSM 880 (Carl Zeiss, Germany). eYFP was excited at 514 nm (5.50%) and detected in the range of 520–610 nm; Phenolic compounds were excited at 405 nm (1.10%) and detected in the range of 200–450 nm to visualize xylem structure and collenchyma. Confocal stacks were acquired with a Z‐step of 50–80 μm using the objectives C‐Apochromat 40x/1.2 W Korr FCS M27.

#### Spray‐inoculation

*Pph* RJ3 p_nptII_::lux p_A1/04/03_::eYFP (Supporting Information Table S1) was incubated prior to inoculation as described above. The bacterial suspension (5 × 10^7^ CFU ml^−1^) was deposited on restricted abaxial areas of the first true leaves of TG plants by spraying the resuspended bacterial culture through a round aperture (about 1 cm^2^) cut into a folded aluminium foil. When spraying, close contact between leaves and aluminium foil ensured that there was no contamination of adjacent areas of the leaf. Four inoculated plants were placed inside a closed transparent box (30 × 20 × 30 cm) with two Oasis flower foams (Oasis Floral Products, UK) previously soaked in water. Plants were incubated for 3 days at 22°C and artificial light was maintained for 16 h within the 24‐h cycle. Negative controls were represented by leaves inoculated solely with 10 mM MgCl_2_.

After 3 days, leaves were detached and placed on square Petri dishes. Leaf petioles were wrapped in water‐soaked cotton discs to allow plant transpiration. Leaves were incubated in the dark for 10 min before imaging with the nightOWL LB 983 *in vivo* imaging system (Berthold Technologies) at 20 min exposure. Bacterial bioluminescence was used to visualize *Pph* localisation and to identify portions of leaves to be imaged with confocal laser scanning microscopy. Leaf vasculatures showing *Pph* colonization were cross‐sectioned by hand‐sectioning (50–80 μm) or imaged as part of leaf discs (50 mm^2^). Samples were imaged with a confocal laser scanning microscope Zeiss LSM 880 (Carl Zeiss).

eYFP was excited at 514 nm (5.50%) and detected in the range of 520–610 nm; phenolic compounds were excited at 405 nm (1.10%) and detected in the range of 200–450 nm to visualize xylem structure; and chlorophyll was excited at 514 nm (5.50%) and detected in the range of 650–720 nm.

Confocal stacks were acquired with a Z‐step of 50–80 μm (for cross sections) or 10–30 μm (for leaf discs) using the objectives LD LCI Plan‐Apochromat 25x/0.8 Imm Korr DIC M27. Leaf volume‐rendering and three‐dimensional models of the confocal stacks were created with the software Imaris 8 (Bitplane AG, Zürich, Switzerland).

### Bacterial bioluminescence assays to detect the effect of plant immune responses on bacterial growth and viability

*Pph* 1302A Δ*hrpA* (this study), *Pph* 1302A Δ*PphB* (this study) and *Pph* 1302A Δ*xerC* (Lovell *et al*., 2009) were tagged using pRS‐p_OXB13_::lux (Table 1; Supporting Information Table S1) as described above. Bacterial cultures were inoculated from frozen glycerol stock onto Luria Bertani (LB) (Sambrook *et al*., 1989) agar (1.5% agar) and grown for 24 h at 28°C. Single colonies were used to inoculate 10 ml LB cultures, which were incubated at 28°C with shaking at 200 rpm. After 24 h, 500 μl pre‐culture was used to set up an overnight 10 ml LB culture. Cultures were centrifuged at 4000*g* for 6 min and resuspended in 10 ml 10 mM MgCl_2_ (10^6^ CFU ml^−1^). Eight plants per bacterial culture were used for the experiment.

The optimal number of replicates were calculated with a jacknife resampling (Hanna, 1989) derived approach before performing the experiment. Briefly, the first two fully developed leaves of 12 two‐week‐old plants (*P. vulgaris* cultivar TG) were syringe‐infiltrated with *Pph* 1302A Δ*PphB* p_OXB13_::lux (10^6^ CFU ml^−1^) (Supporting Information Table S1). Each leaf was infiltrated six times in six small areas (50–80 mm^2^). The six spots within the same leaf were considered technical replicates and were averaged for the calculation of the optimal sample size. Leaves were detached and incubated in the dark for 10 min before imaging with the nightOWL LB 983 *in vivo* imaging system (Berthold Technologies) at 2‐min exposure. Luminescence values were recorded and used for the calculation of the optimal sample size. A custom R script (Supporting Information File S2) was used to randomly sample 1000 thousand times the generated data (12 values, in this case) with a jackknife approach. Standard deviation (SD) for each 1000 resampling per each sample size (from 12 to 1) was used to assess the difference in SD between sample sizes (12‐*n*), where *n* is the sample size. The process was repeated 300 times and statistically significance differences of differences in SD between sample sizes were assessed with Benjamin–Hochberg (BH) post hoc test. Eight was assessed to be the optimal sample size as the reduction in SD from sample size 8 to sample size 9 was not statistically significant.

Subsequently, 2‐week‐old TG plants were syringe‐infiltrated with p_OXB13_::*lux* expressing *Pph* 1302A Δ*hrpA*, *Pph* 1302A Δ*PphB* and *Pph* 1302A Δ*xerC* bacterial cultures (Supporting Information Table S2). The experiment was conducted according to a randomized complete block design (RCBD) (Liu and Berger, 2014) in which the block corresponded to the inoculation time. This was done to account for the passage of time from the first plant inoculations to the last plant inoculations (about 4 h). At time 1, three plants (two leaves per plant) were syringe‐infiltrated with the corresponding bacterial cultures. One leaf per plant was detached and incubated in the dark for 10 min before imaging with the nightOWL LB 983 *in vivo* imaging system (Berthold Technologies) at 2‐min exposure. Luminescence and area of each spot within leaves were recorded. Plants were then incubated for 2 days at 22°C and artificial light was maintained for 16 h within the 24‐h cycle. The same procedure was repeated for subsequent time points. Within each block (time point and tray) plants were randomized. The same bacterial cultures were used for all time points. Negative controls were represented by plants infiltrated with 10 mM MgCl_2_ and infiltrated with the non‐tagged *Pph* 1302A Δ*xerC* bacterial culture to detect any bioluminescence associated with induction of plant immune responses (Bennett *et al*., 2005).

After 2 DPI, luminescence and the area where luminescence is detected were measured as described previously, for the remaining leaves. Exposure time was set at 5 min. Immediately after measuring bioluminescence signal, leaves were used to perform colony counts. Briefly, six leaf discs per leaf were detached from the six inoculated spots with a 1‐cm^2^ cork and pooled together. Samples were ground with a TissueLyser (Qiagen) in 1 ml 10 mM MgCl_2_, and serial dilutions (20 μl drops) were spotted onto LB agar supplemented with gentamicin 10 μg ml^−1^ and nitrofurantoin 25 μg ml^−1^. Plates were incubated at 28°C for 3 days.

### Data analysis and statistics

Processing of data was performed using R (RDC Team (R Development Core Team), 2006) version 3.5.3 and ggplot2 (Wickham, 2016). Requirements of linear models, namely normal distribution of the residuals and homogeneity of variance were assessed using diagnostic plots (Harrison *et al*., 2018). When diagnostic plots were not clearly interpretable, we used Leven's test to assess the homogeneity of variance. Independence of the observations was guaranteed by the experimental design.

## Author contributions

R.S., N.S., and G.M.P. designed the study and the experiments. R.S. performed the experiments and analysed the data. M.B.P. performed the NADP+ assay and discussed the experiments with R.S, I.B. and W.H. contributed to the design for pRS p_nptII_::ilux and I.B. also contributed to the design of the mini‐Tn7 library. R.S. and G.M.P. wrote the manuscript.

## Supporting information


**Supplementary Fig. 1** Expression of FMN reductase (*frp*) increases luminescence in *P. fluorescens* NZ011 when combined with the *lux* operon. *Acinetobacter* strains: *A. baylyi* ADP1 (pIJ11282), *A. baylyi* ADP1 (pRSJ‐p_nptII_::ilux), *A. baylyi* ADP1 (pRSJ‐p_nptII_::lux‐frp). *Pseudomonas fluorescens* strains: *P. fluorescens* NZ011 (pIJ11282), *P. fluorescens* NZ011 (pRSJ‐p_nptII_::ilux), *P. fluorescens* NZ011 (pRSJ‐p_nptII_::lux‐frp). *Pseudomonas syringae* pv. *phaseolicola* (*Pph*) strains: *Pph* 1302A (pIJ11282), *Pph* 1302A (pRSJ‐p_nptII_::ilux), *Pph* 1302A (pRSJ‐p_nptII_::lux‐frp). Plasmids pIJ11282, pRSJ‐p_nptII_::ilux and pRSJ‐p_nptII_::lux‐frp have the same backbone (pIJ11282) and promoter (p_nptII_). **A**: Normalized luminescence of reporter strains in KB (King's B medium) and M9 (M9 minimal medium). **B**: Growth of reporter strains in KB and M9. OD = optical density at 600 nm. Error bar, +/− SE. n = 3.
**Supplementary Fig**. **2**. Expression of *frp* in *P. fluorescens* NZ011 (pRSJ‐p_nptII_:lux‐frp) results in an increase in NADP+ concentration. Lux: *P. fluorescens* NZ011 (pIJ11282). Lux‐frp: *P. fluorescens* NZ011 (pRSJ‐p_nptII_:lux‐frp). NADP+ concentration refers to 25 ul of bacterial culture, for which a 10‐fold dilution was measured as having an optical density (OD600) of 0.33. Error bar +/− SE. Significant differences (t‐test, p < 0.05) are indicated by asterisks. n = 6.
**Supplementary Fig**. **3**. The *lux‐*eYFP operon constructed in this study. **A**: Schematic representation of the lux‐eYFP construct made in this study. p1: p_nptII_, p_OXB20_, p_OXB16_, p_OXB13_, p_OXB11_. p2: p_A1/04/03_, p_lac_. The red ‘T’ represents the T0 terminator. **B**: *Pseudomonas* syringae pv*. phaseolicola* RJ3 (*Pph* RJ3) p_nptII_::lux‐p_A1/04/03_::eYFP imaged with the Typhoon scanner (Amersham/GE Healthcare, UK) using Cy3 settings. eYFP expression is detected. C: *Pph* RJ3 p_nptII_::lux p_A1/04/03_::eYFP imaged with the nightOWL LB 983 at 10 s exposure. Bioluminescence is detected.
**Supplementary Fig**. **4**. **A**: Schematic representation of the pRS‐p_OXB20(1)_::lux plasmid. pRS‐p_OXB20(1_)::lux is derived from pUC18‐mini‐Tn7T‐Gm‐lux. MCS: Long multiple cloning site (490 bp) of pUC18‐mini‐Tn7T‐Gm‐lux plasmid. RBS1: Ribosome Binding site of plasmid pUC18‐mini‐Tn7T‐Gm‐lux. **B**: Schematic representation of the pRS‐_pOXB20_::lux plasmid. RBS2: Ribosome‐binding site of plasmid pIJ11282. The RBS strength was calculated with the RBS calculator (Farasat *et al*., 2014; Ng *et al*., 2015) considering a total DNA length of 45 bp from the starting codon of luxC.
**Supplementary Fig**. **5**. pRS‐p_OXB20_::lux generates increased luminescence compared to pRS‐p_OXB20(1)::_lux in *Pseudomonas syringae* pv. *phaseolicola* 1302A. Plasmid pRS‐p_OXB20(1)_::lux was derived from the existing mini‐Tn7 plasmid pUC18‐mini‐Tn7T‐Gm‐lux (Choi *et al*., 2005) by inserting oriT and ligating the OXB20 promoter upstream of *luxC*. Plasmid pRS‐p_OXB20_::lux has a stronger RBS (derived from pIJ11282) upstream of *luxC* compared to pUC18‐mini‐Tn7T‐Gm‐lux. **A**: Normalized luminescence of *Pph* reporter strains in KB (King's B medium) and M9 (M9 minimal medium). **B**: Growth of reporter strains in KB and M9. OD = optical density at 600 nm. Error bar +/− SE. n = 3
**Supplementary Fig**. **6**. *Pseudomonas syringae* pv. *phaseolicola* 1302A (*Pph* 1302A) reporter strains exhibit growth curves similar to *Pph* 1302A in both rich and minimal medium while providing a wide range of luminescence. Strains: *Pph* 1302A, *Pph* 1302A p_OXB11_::lux‐p_A1/04/03_::eYFP, *Pph* 1302A p_OXB13_::lux‐p_A1/04/03_::eYFP, *Pph* 1302A p_OXB16_::lux‐p_A1/04/03_::eYFP, *Pph* 1302A p_OXB20_::lux‐p_A1/04/03_::eYFP and *Pph* 1302A p_nptII_::lux‐p_A1/04/03_::eYFP. **A**: Normalized luminescence of *Pph* reporter strains in KB (King's B medium) and M9 (M9 minimal medium) **B**: Growth of *Pph* 1302A reporter strains compared to *Pph* 1302A in KB and M9. OD = optical density at 600 nm.. Error bar +/− SE. n = 3.
**Supplementary Fig**. **7**. Strong expression of the *lux* cassette has a small but significant effect on *Pph* 1302A bacterial growth both *in vitro* and *in planta*. **A**: OD values at 3 h of data presented in Supplementary Fig. 6. **nptII**: *Pph* 1302A p_nptII_::lux‐p_A1/04/03_:::eYFP; **OXB20**: *Pph* 1302A p_OXB20_::lux‐p_A1/04/03_::eYFP; **OXB16**: *Pph* 1302A p_OXB16_::lux‐p_A1/04/03_::eYFP; **OXB13**: *Pph* 1302A p_OXB13_::lux‐p_A1/04/03_::eYFP; **OXB11**: *Pph* 1302A p_OXB11_::lux‐p_A1/04/03_::eYFP; **1302A**: *Pph* 1302A. Error bar +/− SE. Significant differences (Tukey HSD, p < 0.05) are indicated by letters. n = 3. **B**: *In planta* growth of *Pph* 1302A reporter strains. TG leaves were syringe infiltrated with *Pph* 1302A reporter strains at 5x10^6^ CFU ml^−1^ in 10 mM MgCl_2_. **nptII**: *Pph* 1302A p_nptII_::lux‐p_A1/04/03_:::eYFP; **OXB16**: *Pph* 1302A p_OXB16_::lux‐p_A1/04/03_::eYFP; **OXB11**: *Pph* 1302A p_OXB11_::lux‐p_A1/04/03_::eYFP; **1302A**: *Pph* 1302A. Error bar +/− SE. Significant differences (Tukey HSD, p < 0.05) are indicated by letters. n = 4.
**Supplementary Fig**. **8**. Detection limit for bioluminescent *Pseudomonas syringae* pv*. phaseolicola* RJ3 (*Pph* RJ3) in agar plates. *Pph* RJ3 p_nptII_::lux‐p_A1/04/03_::eYFP was spotted in serial dilution on an LB agar plate. Images were taken after 1 h using the nightOWL LB 983 at 20 min exposure. Approximately 100 cells/mm^2^ can be detected. Colour scales have different minimum values to avoid oversaturation. CFU/mm^2^ was estimated by colony counting. SE for CFU and SE for luminescence constitute on average 27.7% and 2.66% of the means respectively. n = 3.
**Supplementary Fig**. **9**. *Pseudomonas syringae* pv. *phaseolicola* RJ3 (*Pph* RJ3) colonizes the leaf vasculature of *P. vulgaris* cultivar TG following spray inoculation onto the leaf surface. **A, B**: *Pph* RJ3 p_nptII_::lux‐p_A1/04/03_::eYFP was sprayed in a localized area on the abaxial surface of *Phaseolus vulgaris* cv. Tendergreen leaves through an aperture in an aluminium foil (white dotted line). Images were taken after 2 DPI with the nightOWL LB 983 at 20 min exposure. Red circle indicates leaf discs used for confocal microscopy imaging (C). Red lines indicates cross sections used for confocal microscopy imaging (D,E). **C**: Maximum projections of the leaf vasculature. *Pph* RJ3 p_nptII_::lux‐p_A1/04/03_::eYFP colonies (yellow; white arrow). Chlorophyll (red; black arrow). **D**: image of vasculature cross section. *Pph* RJ3 p_nptII_::lux‐p_A1/04/03_::eYFP colonies (yellow). Xylem autofluorescence (grey). Red circular line indicates area zoomed in panel F. **E**: Maximum projection of vasculature cross section. Xylem autofluorescence (grey). Red circular line indicates area modelled in panel G. **F**: zoom of panel D. *Pph* RJ3 p_nptII_::lux‐p_A1/04/03_::eYFP colonies (yellow; white arrow). Xylem autofluorescence (grey). **G**: Three‐dimensional model of panel E (Imaris 9 Bitplane AG, Zürich, Switzerland). *Pph* RJ3 p_nptII_::lux‐p_A1/04/03_::eYFP colonies (yellow; white arrow). **H**: Three‐dimensional model of panel C (Imaris 9 Bitplane AG, Zürich, Switzerland). Co‐localization of chlorophyll signal (red) and eYFP (yellow), indicates endophytic localization of *Pph* RJ3 p_nptII_::lux‐p_A1/04/03_::eYFP in the leaf vasculature. Scale bars: 40 μm.
**Supplementary Fig**. **10**. *Phaseolus vulgaris* cultivar Tendergreen leaves do not display background bioluminescence or fluorescence under conditions used to image luminescence or eYFP. **A**: nightOWL LB 983 image. The image is automatically generated by the nightOWL LB 983 by overlaying the image acquired with CCD camera with a photo of the sample. Exposure time 20 min. The leaf was incubated for 10 min in darkness before imaging. Black arrows indicate tissue used for confocal microscopy imaging. **B**: details of the *Phaseolus vulgaris* cultivar TG vascular system. In non‐inoculated plants, eYFP signal is not detectable. Chlorophyll (red), eYFP (yellow). **C**: details of xylem vessels of non‐inoculated plants. eYFP signal is not detectable. Xylem (grey), eYFP (yellow). Settings used were the same as for inoculated plants. Scale bars: 40 μm.
**Supplementary Fig**. **11**. The colony count approach is able to detect the effect of PTI but not ETI on the growth of *Pseudomonas syringae* pv. *phaseolicola* in the early stages of infection (2 DPI). *P. vulgaris* cultivar Tendergreen (TG) leaves were syringe infiltrated with *Pph* 1302A Δ*xerC*, Δ*hrpA* and Δ*PphB* at 10^6^ CFU ml^−1^ in 10 mM MgCl_2_. **A**: Details of the symptoms caused by *Pph* 1302A knock‐out mutants in TG leaves at 2 days post inoculation (DPI). The hypersensitive response (HR) is evident on leaves inoculated with *Pph* 1302A Δ*xerC*. **B**: Colony count data for inoculated leaves at 2 DPI. Error bar +/− SE. Significant differences (Student's T‐test, p < 0.05) are indicated by letters. n = 8.

**Supplementary Video 1** *Pph RJ3‐*eYFP colonizes the collenchyma of the *Phaseolus vulgaris* cultivar Tendergreen (TG) leaf vasculature. *Pph RJ3‐*eYFP (Godfrey *et al*. 2010) was syringe‐infiltrated at 10^6^ CFU ml^−1^ into the first true leaves of TG plants. Leaf disks were detached at 3 days post inoculation and imaged with a TCS Leica SP5 confocal microscope in a xyz series (objective used: HCX PL APO CS 20.0x0.70 IMM UV). eYFP was excited with the 488 nm wave length laser. Cyan (*Pph* RJ3 cells); Red (Chlorophyll autofluorescence); Grey (bright field).

**Supplementary Video 2** *Pph RJ3‐*eYFP (Godfrey *et al*., 2010) moves systemically inside the vasculature of *Phaseolus vulgaris* cultivar Tendergreen (TG). *Pph RJ3‐*eYFP was syringe‐infiltrated at 10^6^ CFU ml^−1^ into the first true leaves of TG plants. Leaf disks were detached at 1 day post inoculation and imaged with the a TCS Leica SP5 confocal microscope in a xyz series (objective used: HC PL APO CS2 63.0x1.20 WATER UV). eYFP was excited with the 488 nm wave length laser. Cyan (*Pph* RJ3 cells); Red (Chlorophyll autofluorescence); Grey (bright field).

**Supplementary Table 1** Plasmids used in this study.
**Supplementary Table 2**. List of strains used
**Supplementary Table 3**. List of primers used for cloning
**Supplementary Table 5**. List of primers used to sequence eYFP and *frp*.
**Supplementary Table 6**. List of primers used to sequence the *lux* operon

**Appendix S1.** Supporting Information.

**Appendix S2.** Supporting Information.

## Data Availability

The research materials supporting this publication have been deposited in the Oxford Research Archive (ORA) as https://doi.org/10.5287/bodleian:y0r8ErQja and https://doi.org/10.5287/bodleian:KOeJ8agdn. If you wish to access the biological material that is described in this study please contact gail.preston@plants.ox.ac.uk.
