# Supplementary material for: From macro to micro: a combined bioluminescence‐fluorescence approach to monitor bacterial localization
Source: Environ Microbiol. 2021 Jan 22;23(4):2070–85. doi: 10.1111/1462-2920.15296 (PMC8614114; doi:10.1111/1462-2920.15296)

Supplementary Figure 1.


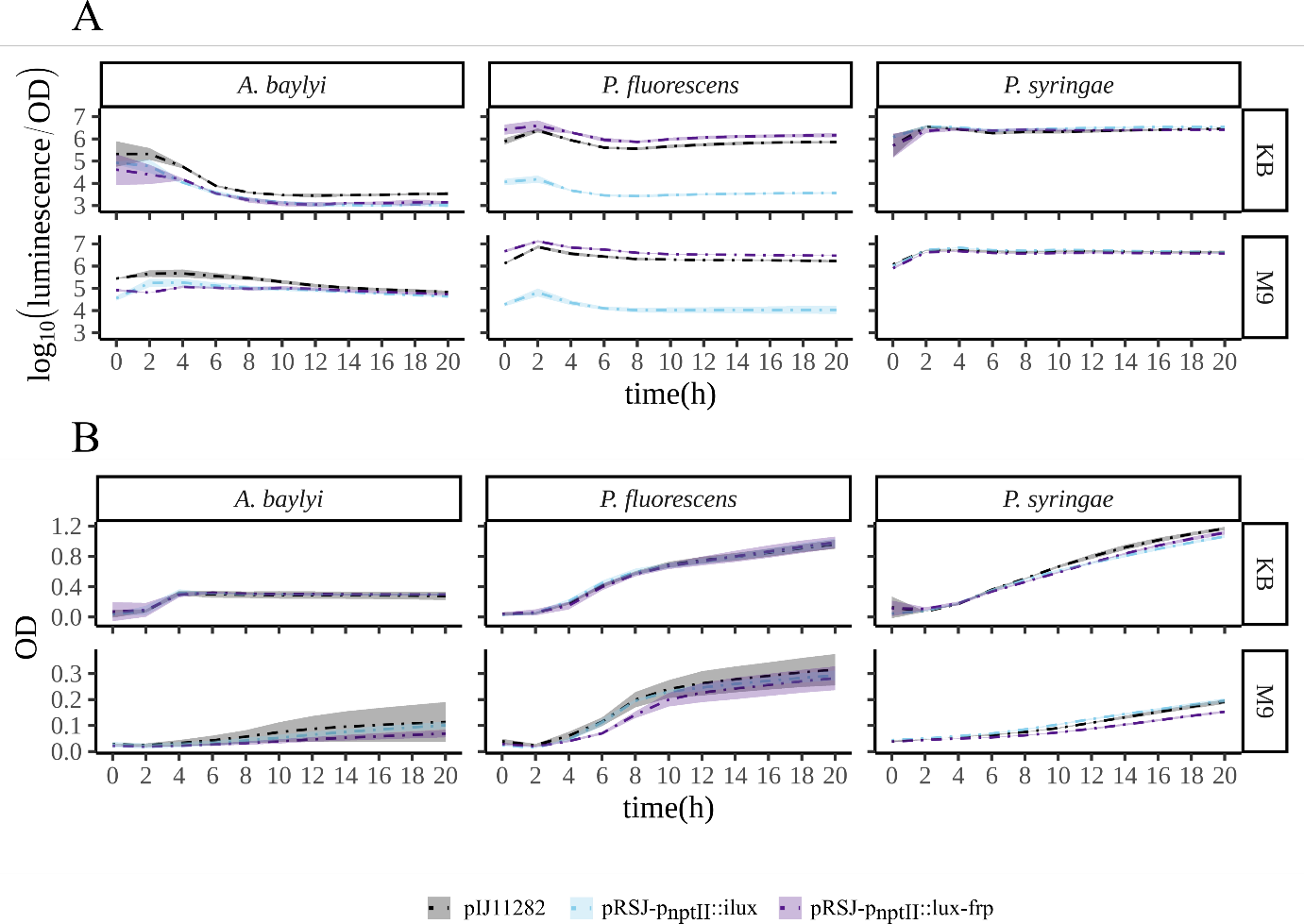


Supplementary Figure 2.


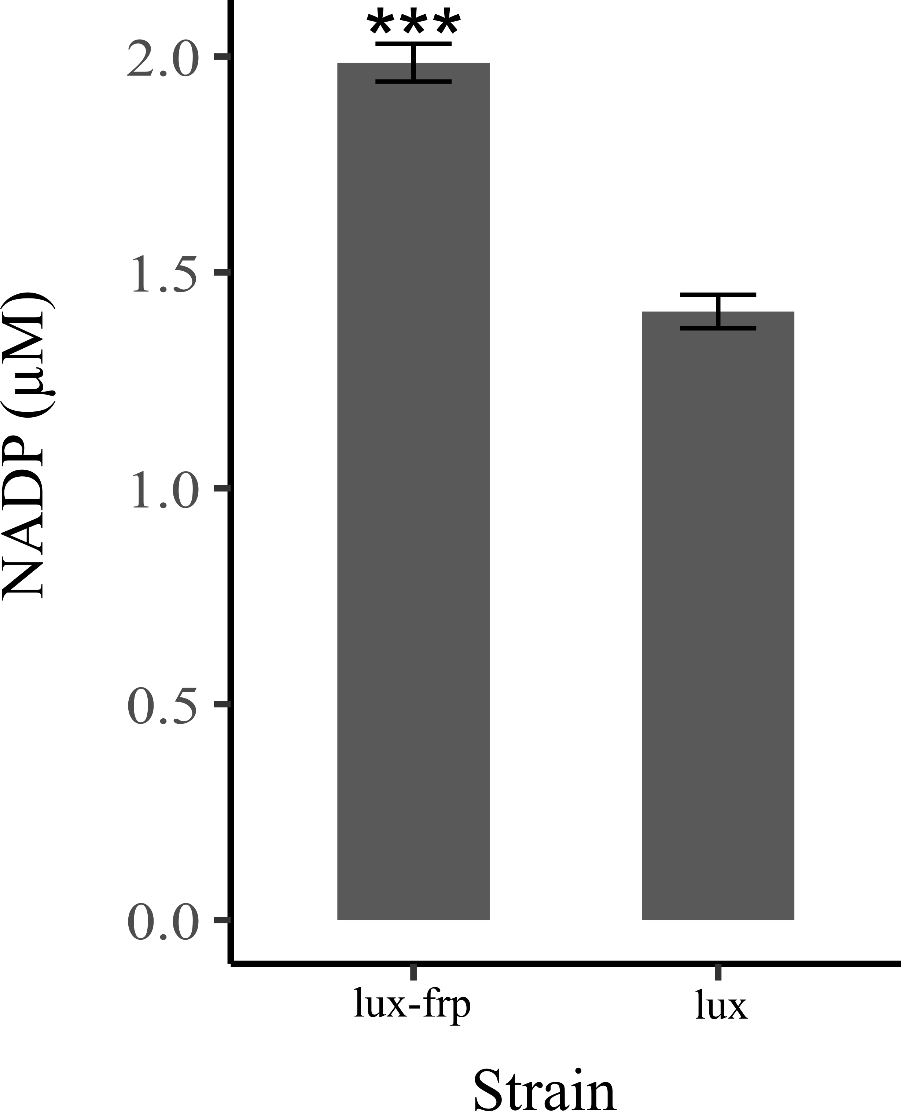


Supplementary Figure 3.


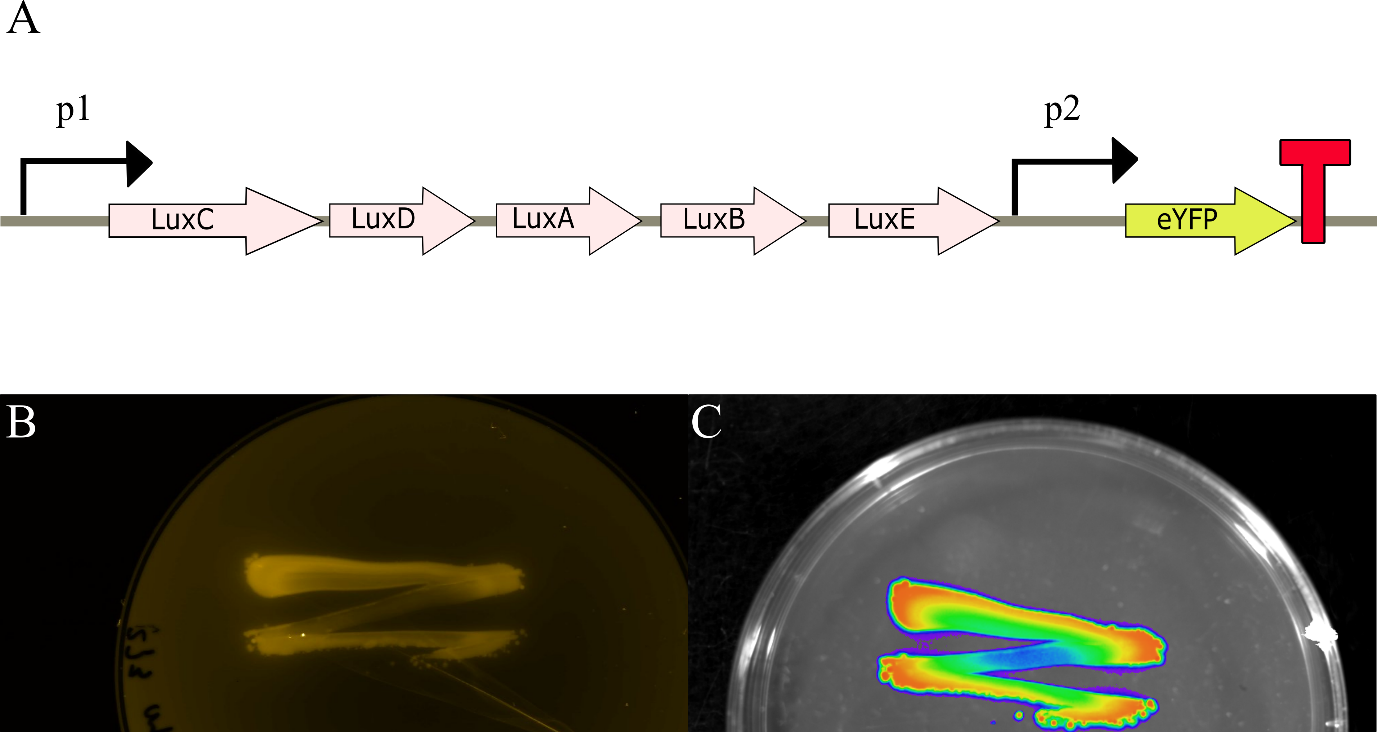


Supplementary Figure 4.


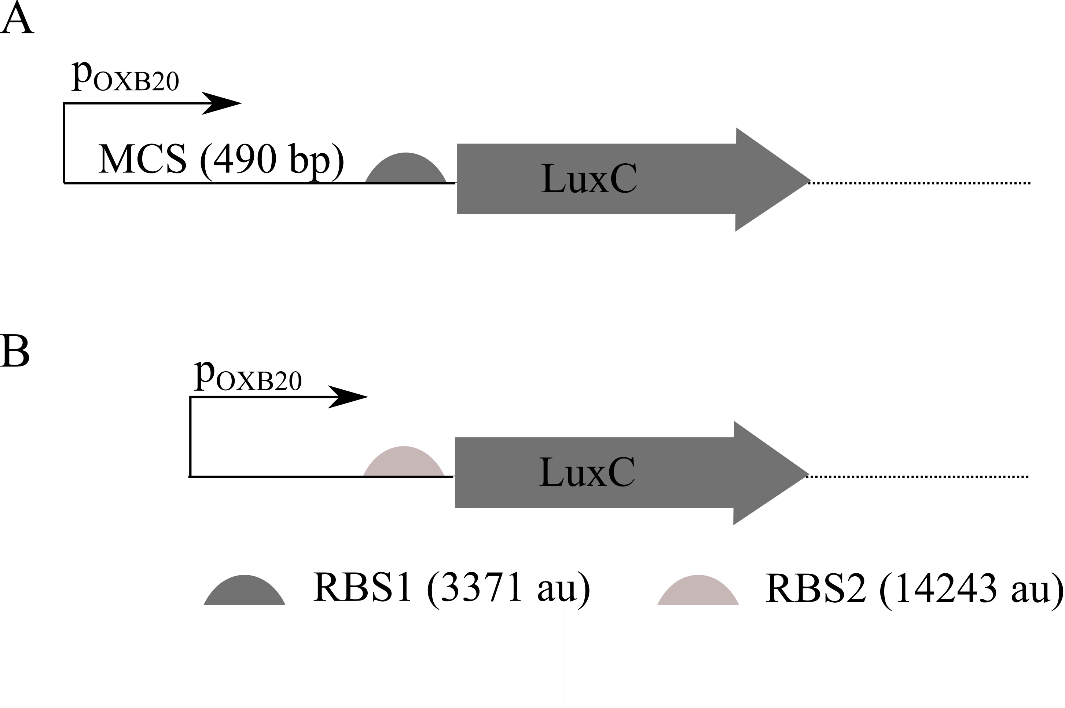


Supplementary Figure 5.


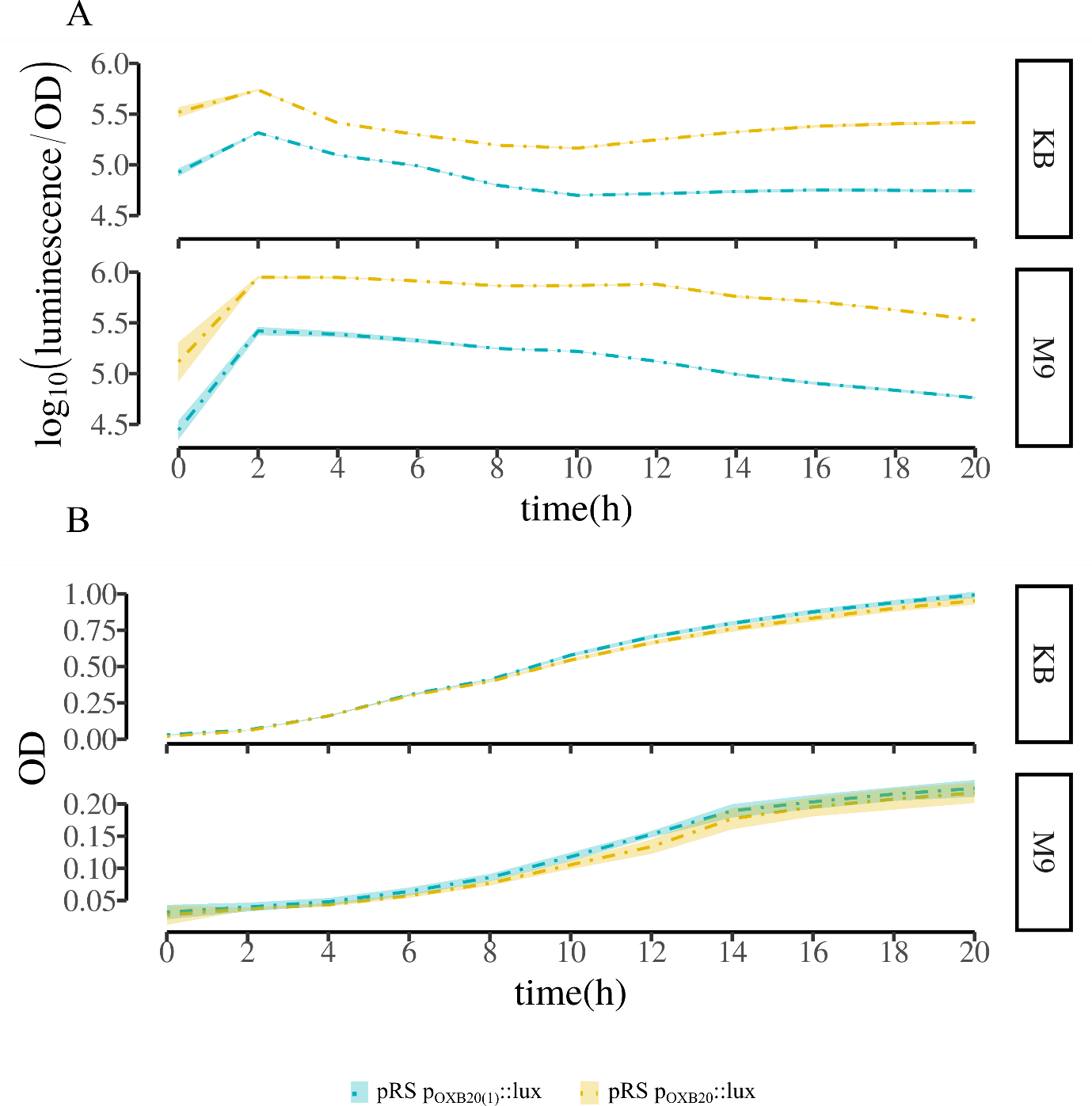


Supplementary Figure 6


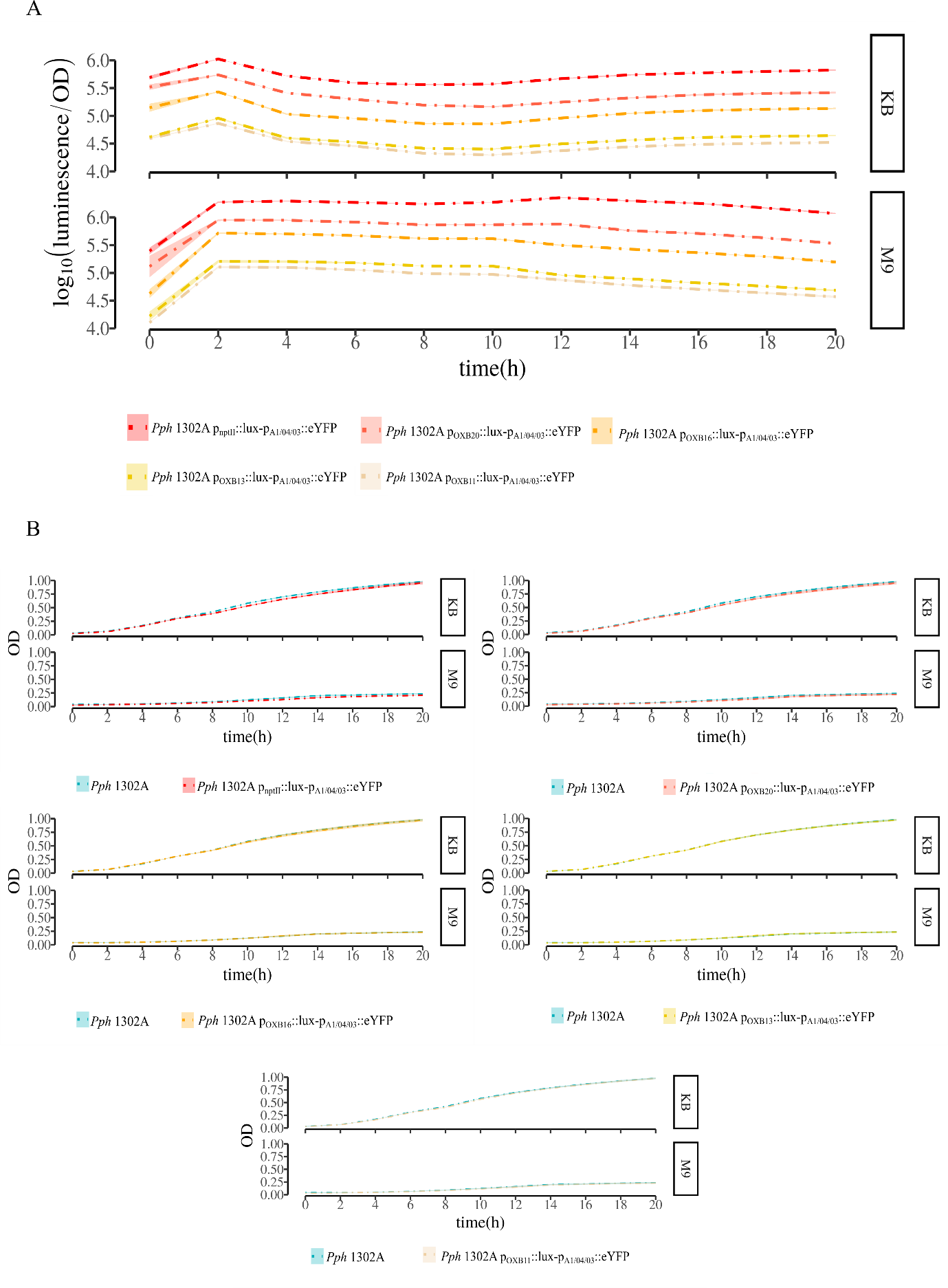


Supplementary Figure 7


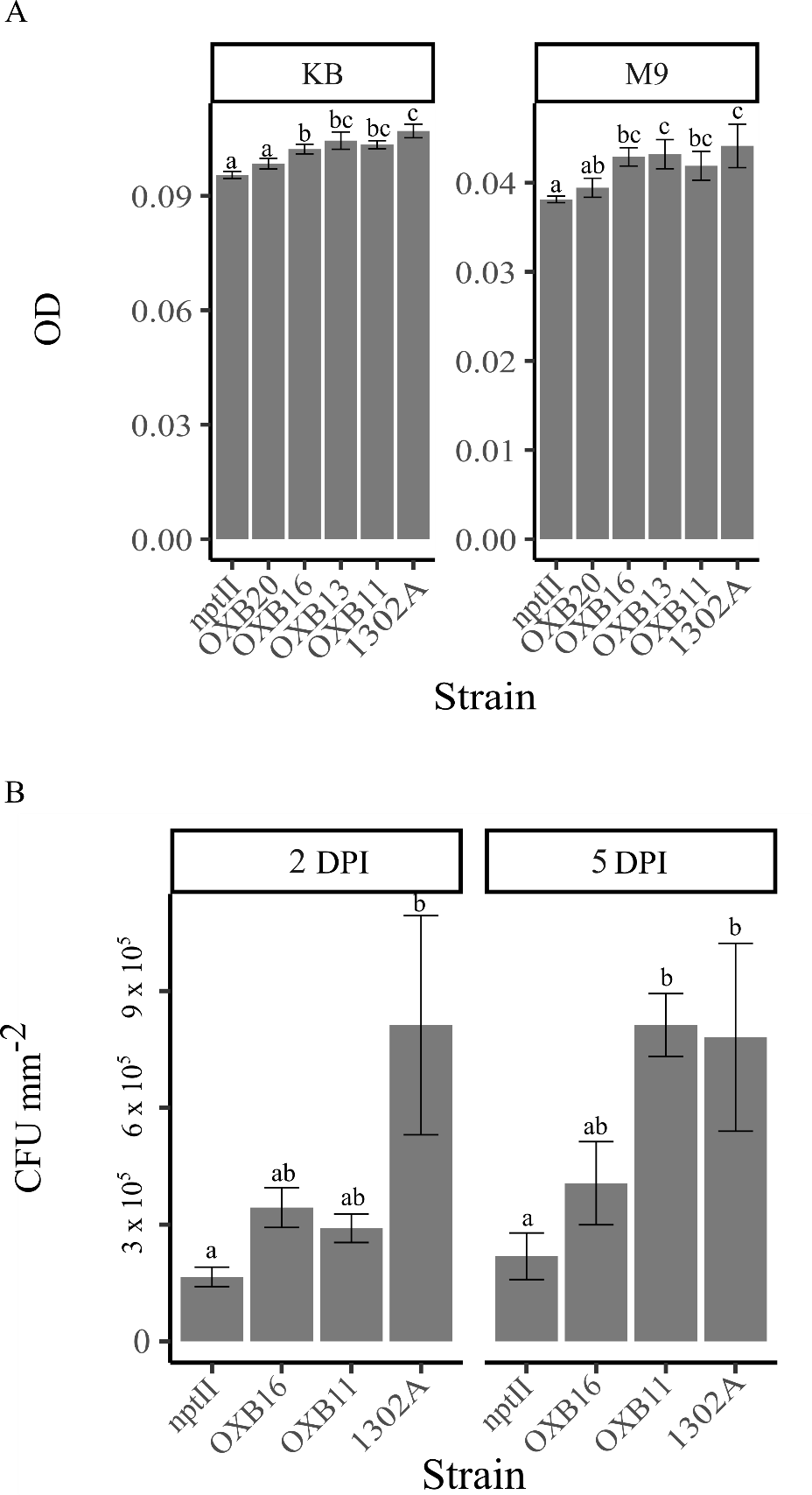


Supplementary Figure 8.


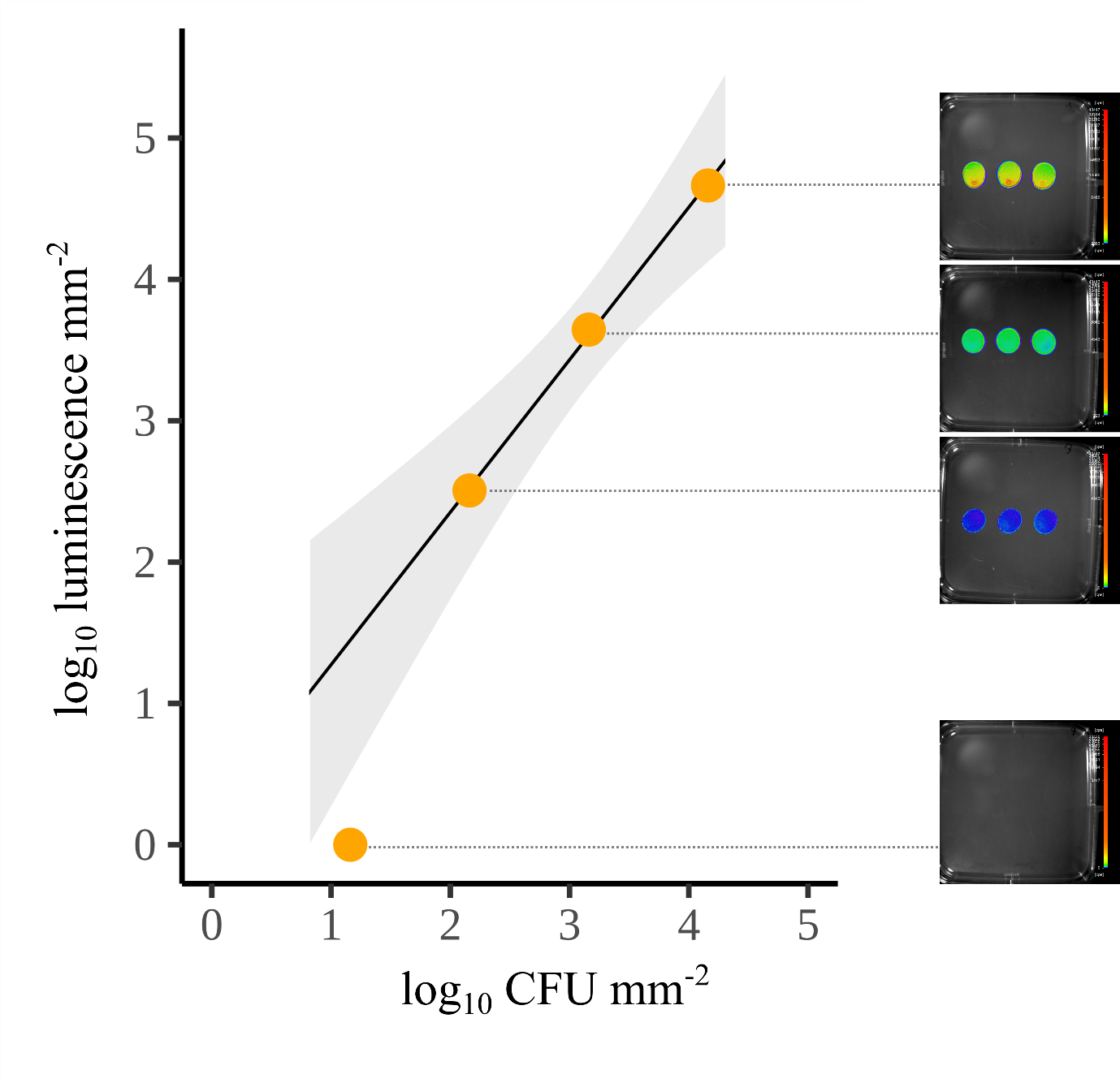


Supplementary Figure 9.


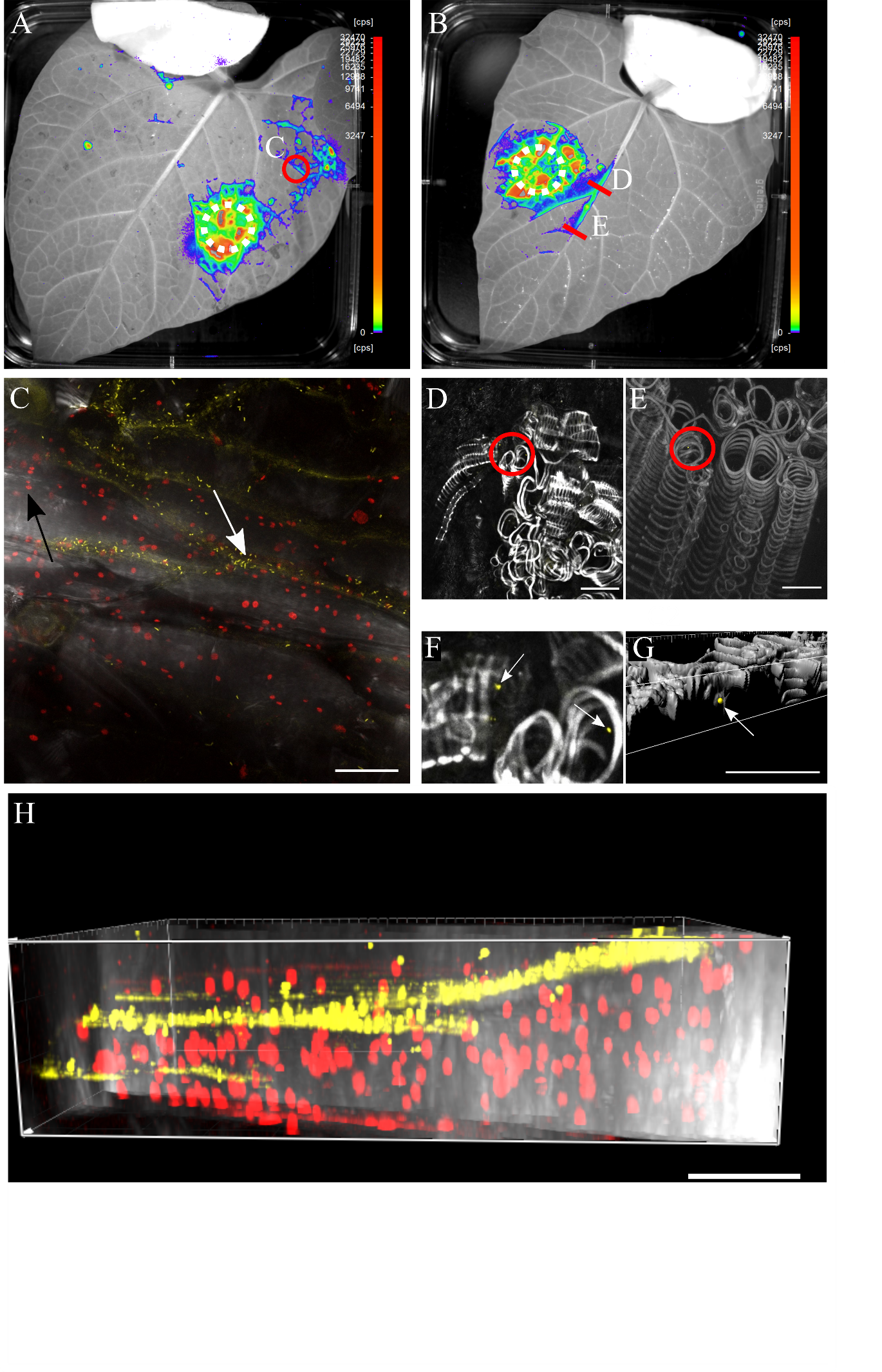


Supplementary Figure 10


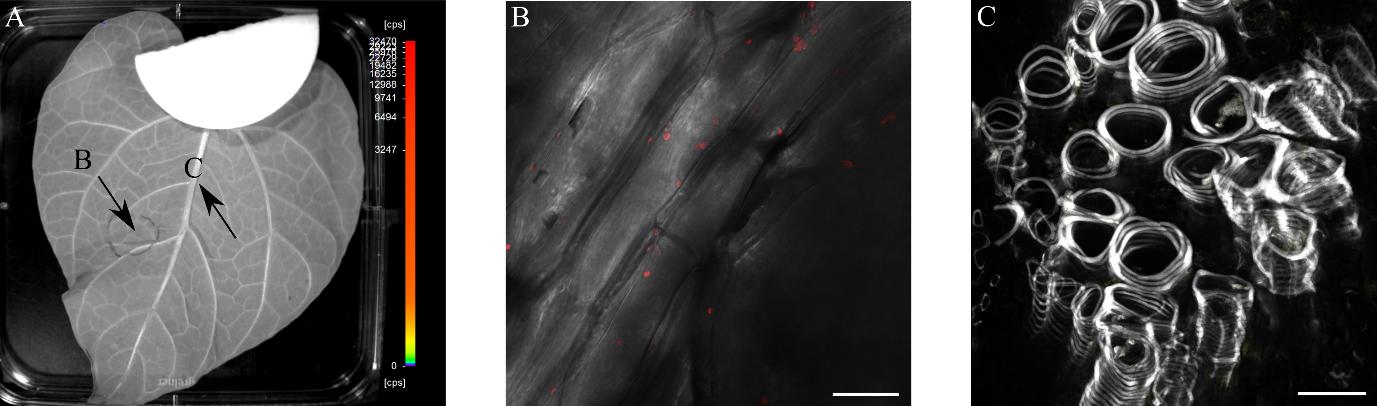


Supplementary Figure 11


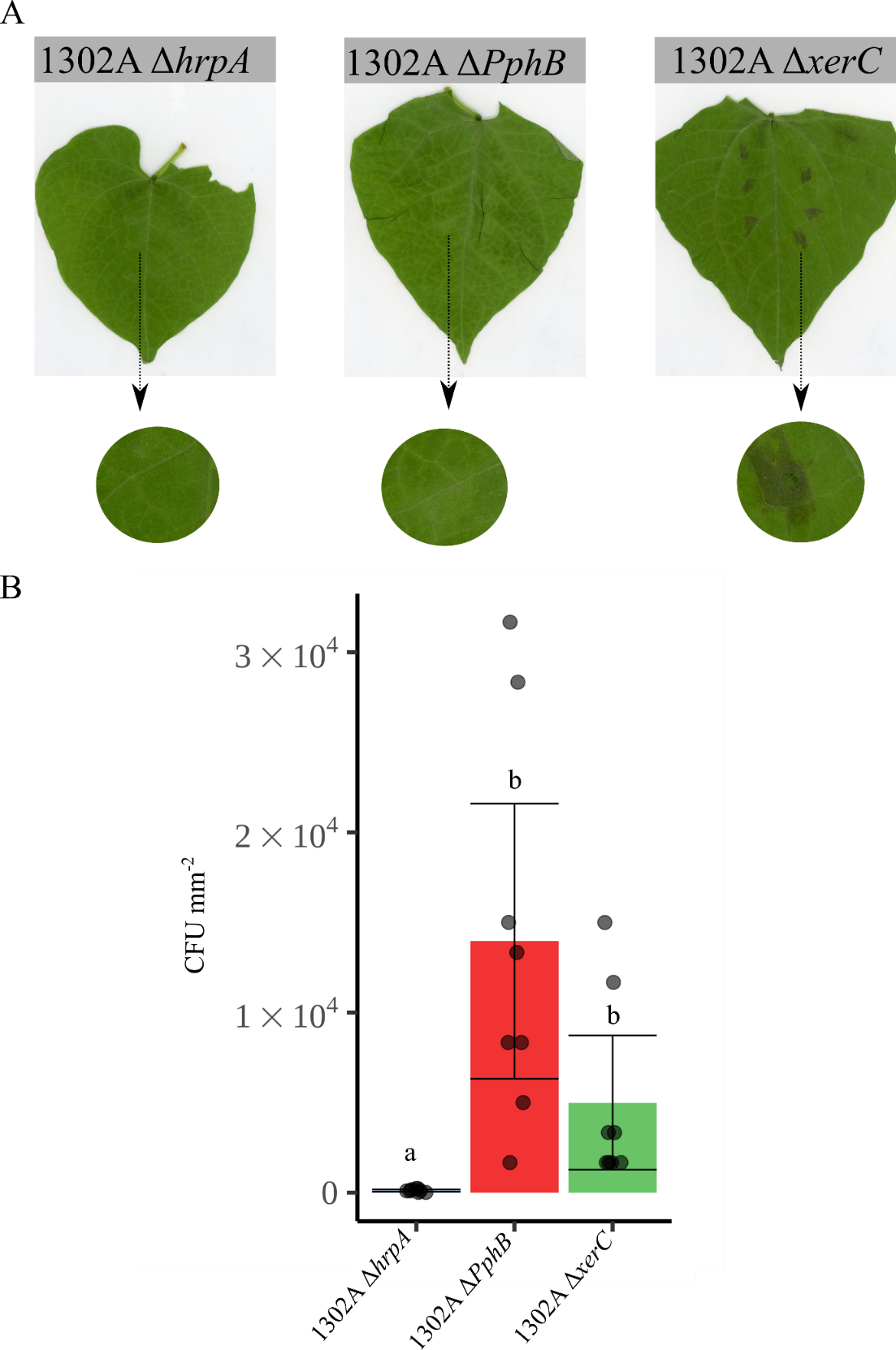

Supplement: Supplementary file 1 — Supplementary Fig. 1 Expression of FMN reductase (frp) increases luminescence in P. fluorescens NZ011 when combined with the lux operon. Acinetobacter strains: A. baylyi ADP1 (pIJ11282), A. baylyi ADP1 (pRSJ‐pnptII::ilux), A. baylyi ADP1 (pRSJ‐pnptII::lux‐frp). Pseudomonas fluorescens strains: P. fluorescens NZ011 (pIJ11282), P. fluorescens NZ011 (pRSJ‐pnptII::ilux), P. fluorescens NZ011 (pRSJ‐pnptII::lux‐frp). Pseudomonas syringae pv. phaseolicola (Pph) strains: Pph 1302A (pIJ11282), Pph 1302A (pRSJ‐pnptII::ilux), Pph 1302A (pRSJ‐pnptII::lux‐frp). Plasmids pIJ11282, pRSJ‐pnptII::ilux and pRSJ‐pnptII::lux‐frp have the same backbone (pIJ11282) and promoter (pnptII). A: Normalized luminescence of reporter strains in KB (King's B medium) and M9 (M9 minimal medium). B: Growth of reporter strains in KB and M9. OD = optical density at 600 nm. Error bar, +/− SE. n = 3. Supplementary Fig. 2. Expression of frp in P. fluorescens NZ011 (pRSJ‐pnptII:lux‐frp) results in an increase in NADP+ concentration. Lux: P. fluorescens NZ011 (pIJ11282). Lux‐frp: P. fluorescens NZ011 (pRSJ‐pnptII:lux‐frp). NADP+ concentration refers to 25 ul of bacterial culture, for which a 10‐fold dilution was measured as having an optical density (OD600) of 0.33. Error bar +/− SE. Significant differences (t‐test, p < 0.05) are indicated by asterisks. n = 6. Supplementary Fig. 3. The lux‐eYFP operon constructed in this study. A: Schematic representation of the lux‐eYFP construct made in this study. p1: pnptII, pOXB20, pOXB16, pOXB13, pOXB11. p2: pA1/04/03, plac. The red ‘T’ represents the T0 terminator. B: Pseudomonas syringae pv. phaseolicola RJ3 (Pph RJ3) pnptII::lux‐pA1/04/03::eYFP imaged with the Typhoon scanner (Amersham/GE Healthcare, UK) using Cy3 settings. eYFP expression is detected. C: Pph RJ3 pnptII::lux pA1/04/03::eYFP imaged with the nightOWL LB 983 at 10 s exposure. Bioluminescence is detected. Supplementary Fig. 4. A: Schematic representation of the pRS‐pOXB20(1)::lux plasmid. pRS‐pOXB20(1) [file EMI-23-2070-s003.docx]
