# Supplementary material for: From macro to micro: a combined bioluminescence‐fluorescence approach to monitor bacterial localization
Source: Environ Microbiol. 2021 Jan 22;23(4):2070–85. doi: 10.1111/1462-2920.15296 (PMC8614114; doi:10.1111/1462-2920.15296)
Supplement: Supplementary file 4 — Supplementary Table 1 Plasmids used in this study. Supplementary Table 2. List of strains used Supplementary Table 3. List of primers used for cloning Supplementary Table 5. List of primers used to sequence eYFP and frp. Supplementary Table 6. List of primers used to sequence the lux operon [file EMI-23-2070-s006.docx]

Supplementary Table 1. Plasmids used in this study.

| Plasmid | Relevant characteristics^1^ |
| --- | --- |
| pRS-p_nptII_::lux | Suicide vector for mini-Tn7 delivery. nptII promoter driving expression of *luxCDABE.* Backbone pUC18T, suitable for conjugation. MscI digestion opens the plasmid after *luxE.* PdiI (NaeI)/SnaBI digestion removes nptII promoter. Gm^r^. This study. |
| pRS-p_OXB20_::lux | Suicide vector for mini-Tn7 delivery. OXB20 (*recA* promoter, Oxford Genetics Limited) promoter driving expression of *luxCDABE.* Backbone pUC18T, suitable for conjugation. MscI opens the plasmid after *luxE.* Gm^r^. This study. |
| pRS-p_OXB16_::lux | Suicide vector for mini-Tn7 delivery. OXB16 (modified from *recA* promoter, Oxford Genetics Limited) promoter driving expression of *luxCDABE.* Backbone pUC18T, suitable for conjugation. MscI digestion opens the plasmid after *luxE.* Gm^r^. This study. |
| pRS-p_OXB13_::lux | Suicide vector for mini-Tn7 delivery. OXB13 (modified from *recA* promoter, Oxford Genetics Limited) promoter driving expression of *luxCDABE.* Backbone pUC18T, suitable for conjugation. MscI digestion opens the plasmid after *luxE.* Gm^r^. This study. |
| pRS-p_OXB11_::lux | Suicide vector for mini-Tn7 delivery. OXB11 (modified from *recA* promoter, Oxford Genetics Limited) promoter driving expression of *luxCDABE.* Backbone pUC18T, suitable for conjugation. MscI digestion opens the plasmid after *luxE.* Gm^r^. This study. |
| pRS-p_nptII_::lux-frp | Suicide vector for mini-Tn7 delivery. nptII promoter driving expression of *luxCDABE.* FMN reductase (*frp*) downstream of *luxE*. Backbone pUC18T, suitable for conjugation. MscI digestion opens the plasmid after *frp.* PdiI (NaeI)/SnaBI digestion removes nptII promoter. Gm^r^. This study. |
| pRS-p_OXB20_::lux-frp | Suicide vector for mini-Tn7 delivery. OXB20 (*recA* promoter, Oxford Genetics Limited) promoter driving expression of *luxCDABE.* FMN reductase (*frp*) downstream of *luxE*. Backbone pUC18T, suitable for conjugation. MscI digestion opens the plasmid after *frp.* Gm^r^. This study. |
| pRS-p_nptII_::lux-p_A1/04/03_::eYFP | Suicide vector for mini-Tn7 delivery. nptII promoter driving expression of *luxCDABE* with A1/04/03 promoter driving expression of eYFP after *luxE.* Lambda T0 terminator added downstream of eYFP. Backbone pUC18T, suitable for conjugation. MscI digestion opens the plasmid after lambda T0 terminator*.* PdiI (NaeI)/SnaBI digestion removes nptII promoter. Gm^r^. This study. |
| pRS-p_OXB20_::lux-p_A1/04/03_::eYFP | Suicide vector for mini-Tn7 delivery. OXB20 promoter (*recA* promoter, Oxford Genetics Limited) driving expression of *luxCDABE* with A1/04/03 promoter driving expression of eYFP after *luxE.* Lambda T0 terminator added downstream of eYFP. Backbone pUC18T, suitable for conjugation. MscI digestion opens the plasmid after lambda T0 terminator*.* Gm^r^. This study. |
| pRS-p_OXB16_::lux-p_A1/04/03_::eYFP | Suicide vector for mini-Tn7 delivery. OXB16 (modified from *recA* promoter, Oxford Genetics Limited) promoter driving expression of *luxCDABE* with A1/04/03 promoter driving expression of eYFP after *luxE.* Lambda T0 terminator added downstream of eYFP. Backbone pUC18T, suitable for conjugation. MscI digestion opens the plasmid after lambda T0 terminator*.* Gm^r^. This study. |
| pRS-p_OXB13_::lux-p_A1/04/03_::eYFP | Suicide vector for mini-Tn7 delivery. OXB13 (modified from *recA* promoter, Oxford Genetics Limited) promoter driving expression of *luxCDABE* with A1/04/03 promoter driving expression of eYFP after *luxE.* Lambda T0 terminator added downstream of eYFP. Backbone pUC18T, suitable for conjugation. MscI digestion opens the plasmid after lambda T0 terminator*.* Gm^r^. This study. |
| pRS-p_OXB11_::lux-p_A1/04/03_::eYFP | Suicide vector for mini-Tn7 delivery. OXB11 (modified from *recA* promoter, Oxford Genetics Limited) promoter driving expression of *luxCDABE* with A1/04/03 promoter driving expression of eYFP after *luxE.* Lambda T0 terminator added downstream of eYFP. Backbone pUC18T, suitable for conjugation. MscI digestion opens the plasmid after lambda T0 terminator*.* Gm^r^. This study. |
| pRS-p_nptII_::lux-frp-p_A1/04/03_::eYFP | Suicide vector for mini-Tn7 delivery. nptII promoter driving expression of *luxCDABE-frp* with A1/04/03 promoter driving expression of eYFP after *frp.* Lambda T0 terminator added downstream of eYFP. Backbone pUC18T, suitable for conjugation. MscI digestion opens the plasmid after lambda T0 terminator*.* PdiI (NaeI)/SnaBI digestion removes nptII promoter. Gm^r^. This study. |
| pRS-p_OXB20_::lux-frp-p_A1/04/03_::eYFP | Suicide vector for mini-Tn7 delivery. OXB20 promoter (*recA* promoter, Oxford Genetics Limited) driving expression of *luxCDABE frp* with A1/04/03 promoter driving expression of eYFP after *frp.* Lambda T0 terminator added downstream of eYFP. Backbone pUC18T, suitable for conjugation. MscI digestion opens the plasmid after lambda T0 terminator*.* Gm^r^. This study. |
| pRS-p_nptII_::lux-p_lac_::eYFP | Suicide vector for mini-Tn7 delivery. nptII promoter driving expression of *luxCDABE* with lac promoter driving expression of eYFP after *luxE.* Lambda T0 terminator added downstream of eYFP. Backbone pUC18T, suitable for conjugation. MscI digestion opens the plasmid after lambda T0 terminator*.* PdiI (NaeI)/SnaBI digestion removes nptII promoter. Gm^r^. This study. |
| pRS-p_OXB20_::lux-p_lac_::eYFP | Suicide vector for mini-Tn7 delivery. OXB20 (*recA* promoter, Oxford Genetics Limited) promoter driving expression of *luxCDABE* with lac promoter driving expression of eYFP after *luxE.* Lambda T0 terminator added downstream eYFP. Backbone pUC18T, suitable for conjugation. MscI digestion opens the plasmid after lambda T0 terminator*.* Gm^r^. This study. |
| pRS-p_OXB16_::lux-p_lac_::eYFP | Suicide vector for mini-Tn7 delivery. OXB16 (modified from *recA* promoter, Oxford Genetics Limited) promoter driving expression of *luxCDABE* with lac promoter driving expression of eYFP after *luxE.* Lambda T0 terminator added downstream eYFP. Backbone pUC18T, suitable for conjugation. MscI digestion opens the plasmid after lambda T0 terminator*.* Gm^r^. This study. |
| pRS-p_OXB13_::lux-p_lac_::eYFP | Suicide vector for mini-Tn7 delivery. OXB13 (modified from *recA* promoter, Oxford Genetics Limited) promoter driving expression of *luxCDABE* with lac promoter driving expression of eYFP after *luxE.* Lambda T0 terminator added downstream of eYFP. Backbone pUC18T, suitable for conjugation. MscI digestion opens the plasmid after lambda T0 terminator*.* Gm^r^. This study. |
| pRS-p_OXB11_::lux-p_lac_::eYFP | Suicide vector for mini-Tn7 delivery. OXB11 (modified from *recA* promoter, Oxford Genetics Limited) promoter driving expression of *luxCDABE* with lac promoter driving expression of eYFP after *luxE.* Lambda T0 terminator added downstream of eYFP. Backbone pUC18T, suitable for conjugation. MscI digestion opens the plasmid after lambda T0 terminator*.* Gm^r^. This study. |
| pRS-p_nptII_::lux-frp p_lac_::eYFP | Suicide vector for mini-Tn7 delivery. nptII promoter driving expression of *luxCDABE-frp* with lac promoter driving expression of eYFP after *frp.* Lambda T0 terminator added downstream of eYFP. Backbone pUC18T, suitable for conjugation. MscI digestion opens the plasmid after lambda T0 terminator*.* PdiI (NaeI)/SnaBI digestion removes nptII promoter. Gm^r^. This study. |
| pRS-p_OXB20_::lux-frp-p_lac_::eYFP | Suicide vector for mini-Tn7 delivery. OXB20 promoter (*recA* promoter, Oxford Genetics Limited) driving expression of *luxCDABE-frp* with lac promoter driving expression of eYFP after *frp.* Lambda T0 terminator added downstream of eYFP. Backbone pUC18T, suitable for conjugation. MscI digestion opens the plasmid after lambda T0 terminator*.* Gm^r^. This study. |
| pRS-p_OXB20(1)_::lux | Suicide vector for mini-Tn7 delivery. OXB20 promoter (*recA* promoter, Oxford Genetics Limited) driving expression of *luxCDABE.* Backbone pUC18-mini-Tn7T-Gm-lux with oriT, suitable for conjugation. Gm^r^. This study. |
| pRS-p_OXB20(1)::_lux-p_A1/04/03_::eYFP | Suicide vector for mini-Tn7 delivery. OXB20 promoter (*recA*, Oxford Genetics Limited) driving expression of *luxCDABE* with A1/04/03 promoter driving expression of eYFP after *luxE.* Lambda T0 terminator added downstream eYFP. Backbone pUC18-mini-Tn7T-Gm-lux with oriT, suitable for conjugation. Gm^r^. This study. |
| pRSJ-p_nptII_::ilux | Broad host range, low-copy number plasmid. nptII promoter driving expression of *ilux.* Backbone of pIJ11282 (Frederix *et al.,* 2014). Tet^r^ |
| pRSJ-p_nptII_::lux-frp | Broad host range, low-copy number plasmid. nptII promoter driving expression of *luxCDABE frp.* Backbone of pIJ11282 (Frederix *et al.,* 2014). Tet^r^ |
| pRS-p_lac_::eYFP | Suicide vector for mini-Tn7 delivery. lac promoter driving expression of eYFP. Lambda T0 terminator added downstream of eYFP. Backbone pUC18T, suitable for conjugation. Gm^r^. This study. |
| pIJ11282 | Broad host range, low-copy number plasmid. nptII promoter driving expression of *luxCDBAE.* (Frederix *et al.,* 2014). Tet^r^ |
| pGEX(-) | High-copy number plasmid harbouring *ilux.* (Gregor *et al.,* 2018). Amp^r^ |
| pK18mobsacB | Schäfer *et al.*, (1994). Kan^r^ |
| pUC18T-mini_Tn7T-Gm-dsRedExpress | Choi *et al.*, (2005). Gm^r^ |
| miniTn7(Gm)PA1/04/03-eyfp-a | Klausen *et al.*, (2003). Gm^r^ |
| pUC18-mini-Tn7T-Gm-lux | Choi et al., (2005). Gm^r.^ |
| pUX-BF13 | Miller and Mekalanos, (1988). Carb^r^ |
| pRK2013 | Yoshimoto *et al.*, (1991). Kan^r^ |

^1^ Abbreviations: Gm, gentamicin. Tet, tetracycline. Carb, carbenicillin. Kan, kanamycin. Amp, ampicillin.

Supplementary Table 2. List of strains used

| Strain | Features |
| --- | --- |
| *P. syringae* pv. phaseolicola 1302A (*Pph* 1302°) | Taylor *et al.*, (1996). Carries the genomic island PPHGI-1. Causes disease in *P. vulgaris* cv. Canadian Wonder and induces AvrPphB-mediated effector-triggered immunity (ETI) in *P. vulgaris* cv. Tendergreen. |
| *Pph* RJ3 | Jackson *et al.*, (2000). Derivative of *Pph* that lacks the genomic island PPHGI-1. Causes disease in *P. vulgaris* cv. Canadian Wonder and *P. vulgaris* cv. Tendergreen. |
| *Pph* 1302A Δ*PphB* | *avrPphB* deletion mutant. This study. Causes disease in *P. vulgaris* cv. Canadian Wonder and *P. vulgaris* cv. Tendergreen. |
| *Pph* 1302A Δ*hrpA* | *hrpA* deletion mutant. This study. Lacks a functional type III secretion system and elicits PAMP-triggered immunity (PTI) in *P. vulgaris*. |
| *Pph* 1302A Δ*xerC* | *xerC* mutant. Lovell *et al.*, (2009). Lacks a recombinase (XerC) that acts to excise PPHGI-1. Causes disease in *P. vulgaris* cv. Canadian Wonder and stably elicits ETI in *P. vulgaris* cv. Tendergreen. |
| *Pph* 1302A Δ*PphB* p_OXB13_::lux | *Pph* 1302A Δ*PphB* expressing *lux* under weak constitutive promoter OXB13 (plasmid used: pRS-p_OXB13_::lux). This study. |
| *Pph* 1302A ΔhrpA p_OXB13_::lux | *Pph* 1302A Δ*hrpA* expressing *lux* under weak constitutive promoter OXB13 (plasmid used: pRS-p_OXB13_::lux). This study. |
| *Pph* 1302A ΔxerC p_OXB13_::lux | *Pph* 1302A Δ*xerC* expressing *lux* under weak constitutive promoter OXB13 (plasmid used: pRS-p_OXB13_::lux). This study. |
| *Pph* 1302A p_nptII_::lux-p_A1/04/03_::eYFP | *Pph* 1302A expressing *lux* under constitutive nptII promoter and eYFP under constitutive A1/04/03 promoter (plasmid used: pRS-pnptII::lux-p_A1/04/03_::eYFP). This study. |
| *Pph* 1302A p_OXB20_::lux-p_A1/04/03_::eYFP | *Pph* 1302A expressing *lux* under constitutive OXB20 promoter and eYFP under constitutive A1/04/03 promoter (plasmid used: pRS-pOXB20::lux-p_A1/04/03_::eYFP). This study. |
| *Pph* 1302A p_OXB16_::lux-p_A1/04/03_::eYFP | *Pph* 1302A expressing *lux* under constitutive OXB16 promoter and eYFP under constitutive A1/04/03 promoter. (plasmid used: pRS-pOXB16::lux-p_A1/04/03_::eYFP). This study. |
| *Pph* 1302A p_OXB13_::lux-p_A1/04/03_::eYFP | *Pph* 1302A expressing *lux* under constitutive OXB13 promoter and eYFP under constitutive A1/04/03 promoter. (plasmid used: pRS-pOXB13::lux-p_A1/04/03_::eYFP). This study. |
| *Pph* 1302A p_OXB11_::lux-p_A1/04/03_::eYFP | *Pph* 1302A expressing *lux* under constitutive OXB11 promoter and eYFP under constitutive A1/04/03 promoter. (plasmid used: pRS-pOXB11::lux-p_A1/04/03_::eYFP). This study. |
| *Pph* 1302A p_OXB20(1)_::lux-p_A1/04/03_::eYFP | *Pph* 1302A expressing *lux* under constitutive OXB20 promoter and eYFP under constitutive A1/04/03 promoter (plasmid used: pRS-pOXB20(1)::lux-p_A1/04/03_::eYFP). This study. |
| *Pph* 1302A (pIJ11282) | *Pph* 1302A expressing *lux* under constitutive nptII promoter. This study. |
| *Pph* 1302A (pRSJ-p_nptII_::ilux) | *Pph* 1302A expressing ilux under constitutive nptII promoter. This study. This study. |
| *Pph* 1302A (pRSJ-p_nptII_::lux-frp) | *Pph* 1302A expressing lux frp under constitutive nptII promoter. (plasmid used: pRSJ-p_nptII_::lux-frp). This study. |
| *Acinetobacter baylyi* ADP1 | WT. Metzgar *et al.*, (2004) |
| *Pseudomonas fluorescens* NZ011 | WT. Godfrey *et al.*, (2001) |
| *Acinetobacter baylyi* ADP1 (pIJ11282) | *A. baylyi* ADP1 expressing *lux* under constitutive nptIIpromoter. This study. |
| *Acinetobacter baylyi* ADP1 (pRSJ-p_nptII_::ilux) | *A. baylyi* ADP1 expressing *ilux* under constitutive nptII promoter. This study. |
| *Acinetobacter baylyi* ADP1 (pRSJ-p_nptII_::lux-frp) | *A. baylyi* ADP1 expressing *lux-frp* under constitutive nptII promoter. This study. |
| *Pseudomonas fluorescens* NZ011 (pIJ11282) | *P. fluorescens* NZ011 expressing *lux* under constitutive nptII promoter. This study. |
| *Pseudomonas fluorescens* NZ011 (pRSJ-p_nptII_::ilux) | *P. fluorescens* NZ011 expressing *ilux* under constitutive nptII promoter. This study. |
| *Pseudomonas fluorescens* NZ011 (pRSJ-p_nptII_::lux-frp) | *P. fluorescens* NZ011 expressing *lux-frp* under constitutive nptII promoter. This study. |
| *Pseudomonas fluorescens* NZ011 p_nptII_::lux-frp | *P. fluorescens* NZ011 expressing *lux-frp* under constitutive nptII promoter (plasmid used: pRS-p_nptII_::lux-frp). This study. |
| *Pseudomonas fluorescens* NZ011 p_nptII_::lux | *P. fluorescens* NZ011 expressing *lux* under constitutive nptII promoter (plasmid used: pRS-p_nptII_::lux). This study. |
| *Xanthomonas campestris* 8004 p_OXB20_::lux-p_A1/04/03_::eYFP | *X. campestris* 8004 expressing *lux* under constitutive OXB20 promoter and eYFP under constitutive A1/04/03 promoter (plasmid used: pRS-p_OXB20_::lux-p_A1/04/03_::eYFP). This study. |
| *E. coli DH5*α (pIJ11282) | Frederix *et al.*, (2014) |
| *E. coli* SM10/λ pir (pUX-BF13) | Miller and Mekalanos, (1988) |
| *E. coli* HB101 (pRK2013) | Yoshimoto *et al.*, (1991) |

Supplementary Table 3. List of primers used for cloning

| Primer name | Sequence 5’-3’ |
| --- | --- |
| OXRS_FT | GTAAGGAGAAAATACCGCATCAGGCCGGCCAGCCTCGCAGA |
| OXRS_RT | CAGCCTGAATGGCGAATGGCTTTCCGCTGCATAACCCTG |
| OXRS_F1 | GACGGATCGATCCGGGGAATTCAGGCTTGGAGGATACGTATGACTAAAAAAATTTCATTCATTATTACCGGCCAGGTTG |
| OXRS_R1 | GGGTCAGTTCCGGCTGGGGGTTCAGCAGCCACCTGCAGTTACCTTCTGGCAAGGCCCTTGG |
| OXRS_F2 | CCGAAGCGTTTGATAGTTGATGACCTGCAGTCGACCTAAGGAGAAAGAAATGGTGAAGATACAGCCCATCCCCAC |
| OXRS_R2 | CCTTGTGGGGTCAGTTCCGGCTGGGGGTTCAGCAGCCACCTGCAGCACGTGTTACCTTCTGGCAAGGCCCTTGG |
| OXRS_F3 | TTTTGAAGCTAATTCGATCATGCATGAGGCATTTCACGGACTTTCATGGG |
| OXRS_R3 | ACTAGATTTCACTTATCTGGTTGGCCCCTTGTGGGGTCAGTTCCGG |
| OXRS_F4 | GGCCAACCAGATAAGTGAAATCTAGT |
| OXRS_R4 | TCATGCATGATCGAATTAGCTTCAAAA |
| OXRS_F5 | CCCCCAGCCGGAACTGACCCCACAAGGTGGGTCGACCTAAGGAGAAAGAAATGGTGAAGATACAGCCCATCCCCAC |
| OXRS_R5 | ACTAGATTTCACTTATCTGGTTGGCCATTACCTTCTGGCAAGGCCCTTGG |
| OXRS_F6 | CCGGAACTGACCCCACAAGGTGGCAATACGCAAACCGCCTCTC |
| OXRS_R6 | ACTAGATTTCACTTATCTGGTTGGCCAGAGAGTCATTACCCCAGGCGTT |
| OXRS_F7 | TGAACTCCAAGGGCCTTGCCAGAAGGTAATGGCAATACGCAAACCGCCTCTC |
| OXRS_R7 | TTTGGAACTAGATTTCACTTATCTGGTTGGCCAGAGAGTCATTACCCCAGGCGTT |
| OXRS_F8 | GGAATTGGGTACCTCGCGAAGGCAATACGCAAACCGCCTCTC |
| OXRS_R8 | CACTTATCTGGTTGGCCTGCAAGGCCTGAGAGTCATTACCCCAGGCGTT |
| OXRS_F9 | TACCGCCTTTGAGTGAGCTGATACCGCACTGCGTCAACTGCTCGAA |
| OXRS_R9 | TTACGAAACTCTAAACTCGTTTACGCAGGTGGCCTGCGTACCTATTTTCAT |
| OXRS_F10 | ATGAAAATAGGTACGCAGGCCACCTGCGTAAACGAGTTTAGAGTTTCGTAA |
| OXRS_R10 | TTCCCTTGTCCAGATAGCCCAGTAGCCACAAGGTGTAGCCTGAGCG |
| OXRS_F11 | GCTACTGGGCTATCTGGACAAGGGAA |
| OXRS_R11 | CGGTATCAGCTCACTCAAAGGCGGTA |
| OXRS_F12 | TTGCTCACATGTTCTTTCCTGCG |
| OXRS_R12 | TTCGCTTGCTGTCCATAAAACCG |
| OXRS_F13 | TACCGCCTTTGAGTGAGCTGATACCGATGGATCTTGATGAGGGGTTTGATGA |
| OXRS_R13 | AGAAATCAGAATCGGACGACCGAAGAACTCATGATGTTCATAACGTGATACC |
| OXRS_F14 | GGTATCACGTTATGAACATCATGAGTTCTTCGGTCGTCCAGTTCTGATTTCT |
| OXRS_R14 | TTCCCTTGTCCAGATAGCCCAGTAGCCAACAAGCCACCGAGTAACTGAC |
| OXRS_F15 | AATCCGACCTGACCCTGCT |
| OXRS_R15 | CACGCCCCTCTTTAATACGACGG |

Supplementary Table 4. List of primers used to sequence the *ilux* operon

| Primer name | Sequence 5’-3’ |
| --- | --- |
| OX26 | AATGTCATGCAACCGTAATTCG |
| OX27 | TTCAACACAAATAACGTGGTCG |
| OX28 | TCGACCACGTTATTTGTGTTG |
| OX29 | CAAGCAATTTCTCTGTCTTAAAATCTATTGAG |
| OX30 | ACCAGAAACAGTCAGAGGAG |
| OX31 | CGCTCCTCTGACTGTTTCTG |
| OX32 | CAGGTATGACTTCATATGTTGATAAAC |
| OX33 | ATGCGTCAGCAACCAGTTATC |

Supplementary Table 5. List of primers used to sequence eYFP and *frp*.

| Primer name | Sequence 5’-3’ |
| --- | --- |
| OXRS_S1 | ATGCGTCAGCAACCAGTTATC |
| OXRS_S2 | AACCAGCAACCAGAAGCAGAGTG |

Supplementary Table 6. List of primers used to sequence the *lux* operon

| Primer name | Sequence 5’-3’ |
| --- | --- |
| OX26 | AATGTCATGCAACCGTAATTCG |
| OX36 | CCCACCAAGGTGATACATCACTC |
| OX37_ | TTCTCATGAAAGGCCATCTAAC |
| OX38 | AACGATAATTGGGTCAAGCAAG |
| OX39 | AAAGGACGATTTCGGTTTGG |
| OX40 | ATGCAGGAAATAACGGAGTATG |
| OX41 | TGCTGAAAGATATAAAGCCGTTGC |
| OX42 | CAAGAAATTACAGCAAGCTCAG |
| OX43 | AGATCTTTGTCTTATTGGTTCGCC |

References

Choi, K.-H., Gaynor, J.B., White, K.G., Lopez, C., Bosio, C.M., Karkhoff-Schweizer, R.R., and Schweizer, H.P. (2005) A Tn 7 -based broad-range bacterial cloning and expression system. *Nature Methods* 2: 443.

Frederix, M., Edwards, A., Swiderska, A., Stanger, A., Karunakaran, R., Williams, A., et al. (2014) Mutation of *praR* in *Rhizobium leguminosarum* enhances root biofilms, improving nodulation competitiveness by increased expression of attachment proteins. *Molecular Microbiology* 93: 464–478.

Godfrey, S. a. C., Harrow, S.A., Marshall, J.W., and Klena, J.D. (2001) Characterization by 16S rRNA Sequence Analysis of Pseudomonads Causing Blotch Disease of Cultivated *Agaricus bisporus*. *Appl Environ Microbiol* 67: 4316–4323.

Jackson, R.W., Mansfield, J.W., Arnold, D.L., Sesma, A., Paynter, C.D., Murillo, J., et al. (2000) Excision from tRNA genes of a large chromosomal region, carrying avrPphB, associated with race change in the bean pathogen, *Pseudomonas syringae* pv. *phaseolicola*. *Mol Microbiol* 38: 186–197.

Klausen, M., Heydorn, A., Ragas, P., Lambertsen, L., Aaes‐Jørgensen, A., Molin, S., and Tolker‐Nielsen, T. (2003) Biofilm formation by *Pseudomonas aeruginosa* wild type, flagella and type IV pili mutants. *Molecular Microbiology* 48: 1511–1524.

Lovell, H.C., Mansfield, J.W., Godfrey, S.A.C., Jackson, R.W., Hancock, J.T., and Arnold, D.L. (2009) Bacterial Evolution by Genomic Island Transfer Occurs via DNA Transformation In Planta. *Current Biology* 19: 1586–1590.

Metzgar, D., Bacher, J.M., Pezo, V., Reader, J., Döring, V., Schimmel, P., et al. (2004) *Acinetobacter* sp. ADP1: an ideal model organism for genetic analysis and genome engineering. *Nucleic Acids Res* 32: 5780–5790.

Miller, V.L. and Mekalanos, J.J. (1988) A novel suicide vector and its use in construction of insertion mutations: osmoregulation of outer membrane proteins and virulence determinants in *Vibrio cholerae* requires toxR. *J Bacteriol* 170: 2575–2583.

Schäfer, A., Tauch, A., Jäger, W., Kalinowski, J., Thierbach, G., and Pühler, A. (1994) Small mobilizable multi-purpose cloning vectors derived from the *Escherichia coli* plasmids pK18 and pK19: selection of defined deletions in the chromosome of *Corynebacterium* glutamicum. *Gene* 145: 69–73.

Taylor, J.D., Teverson, D.M., Allen, D.J., and Pastor‐Corrales, M.A. (1996) Identification and origin of races of *Pseudomonas syringae* pv. *phaseolicola* from Africa and other bean growing areas. *Plant Pathology* 45: 469–478.

Yoshimoto, T., Higashi, H., Kanatani, A., Lin, X.S., Nagai, H., Oyama, H., et al. (1991) Cloning and sequencing of the 7 alpha-hydroxysteroid dehydrogenase gene from *Escherichia coli* HB101 and characterization of the expressed enzyme. *Journal of Bacteriology* 173: 2173–2179.
