# Supplementary material for: From macro to micro: a combined bioluminescence‐fluorescence approach to monitor bacterial localization
Source: Environ Microbiol. 2021 Jan 22;23(4):2070–85. doi: 10.1111/1462-2920.15296 (PMC8614114; doi:10.1111/1462-2920.15296)
Supplement: Supplementary file 5 — Appendix S1. Supporting Information. [file EMI-23-2070-s002.docx]

**Mini-Tn7 transformation of *Pseudomonas syringae, Pseudomonas fluorescens* and *Xanthomonas campestris***

The following protocol was used to successfully introduce mini-Tn7 constructs into *Pseudomonas syringae*, *Xanthomonas campestris* and *Pseudomonas fluorescens.* The efficiency of integration varied between strains (10-20 colonies per square petri-dish, 120x120 mm size, for *P. syringae*, 50-100 colonies for *P. fluorescens,* 3-5 colonies for *X. campestris*).

1. Grow a 10-ml culture of *P. syringae* strain in LB medium at 28 ºC with shaking (200 r.p.m) in a sterile 50 ml falcon tube overnight. Moreover, grow overnight 10 ml cultures of *E. coli* strains as follows:

| Strain | Selection |
| --- | --- |
| SM10/λ pir (pUX-BF13) | 50 µg ml^-1^ carbenicillin |
| HB101 (pRK2013) | 20 µg ml^-1^ kanamycin |
| DH5α+ carrying a mobilizable mini-Tn7 vector | 10 µg ml^-1^ gentamicin |

1. Pipette two 1.5 ml aliquots of each culture into two 2 ml microfuge tubes. Centrifuge for 1 minute at room temperature at 8000 r.p.m.
2. Discard the supernatant and resuspend the pellets in 750 µl warm LB (40 ºC). Combine suspensions from the same strain into a single 2 ml microfuge tube.
3. Centrifuge for 1 minute at room temperature at 8000 r.p.m.
4. Discard the supernatant and resuspend the pellets in 1.5 ml warm LB (40 ºC).
5. Centrifuge for 1 minute at room temperature at 8000 r.p.m and repeat step 5.
6. Set up the conjugation mix in a new 1.5 ml microfuge tube as follows: 700 µl recipient, 300 µl donor, 100 µl of each helper.
7. Centrifuge for 1 minute at room temperature at 8000 r.p.m.
8. Discard the supernatant and resuspend the pellets in 25 µl warm LB (40 ºC).
9. Spot the conjugation mix onto a pre-warmed LB (28 ºC) (1.5% agar) plate and incubate for 24 hours at 28 ºC.
10. Resuspend the conjugation mix in 200 µl LB. Spread 200 µl of the conjugation mix onto LB (1.5% agar) supplemented with 10 µg ml^-1^ gentamicin and 25 µg ml^-1^ nitrofurantoin (to select against *E. coli*).
11. Incubate at 28 ºC for up to 4 days.
